# Predicting the Valence of a Scene from Observers’ Eye Movements

**DOI:** 10.1371/journal.pone.0138198

**Published:** 2015-09-25

**Authors:** Hamed R.-Tavakoli, Adham Atyabi, Antti Rantanen, Seppo J. Laukka, Samia Nefti-Meziani, Janne Heikkilä

**Affiliations:** 1 Center for Machine Vision Research, Department of Computer Science and Engineering, University of Oulu, Oulu, Finland; 2 Autonomous Systems and Advance Robotics Research Centre, University of Salford Manchester, Salford, United Kingdom; 3 School of Computer Science, Engineering and Mathematics, Flinders University of South Australia, Adelaide, Australia; 4 Learning Research Laboratory (LearnLab), P.O. Box 2000, 90014 University of Oulu, Oulu, Finland; Bournemouth University, UNITED KINGDOM

## Abstract

Multimedia analysis benefits from understanding the emotional content of a scene in a variety of tasks such as video genre classification and content-based image retrieval. Recently, there has been an increasing interest in applying human bio-signals, particularly eye movements, to recognize the emotional gist of a scene such as its valence. In order to determine the emotional category of images using eye movements, the existing methods often learn a classifier using several features that are extracted from eye movements. Although it has been shown that eye movement is potentially useful for recognition of scene valence, the contribution of each feature is not well-studied. To address the issue, we study the contribution of features extracted from eye movements in the classification of images into pleasant, neutral, and unpleasant categories. We assess ten features and their fusion. The features are histogram of saccade orientation, histogram of saccade slope, histogram of saccade length, histogram of saccade duration, histogram of saccade velocity, histogram of fixation duration, fixation histogram, top-ten salient coordinates, and saliency map. We utilize machine learning approach to analyze the performance of features by learning a support vector machine and exploiting various feature fusion schemes. The experiments reveal that *‘saliency map’*, *‘fixation histogram’*, *‘histogram of fixation duration’*, and *‘histogram of saccade slope’* are the most contributing features. The selected features signify the influence of fixation information and angular behavior of eye movements in the recognition of the valence of images.

## Introduction

The eyes are said to be the “window to the soul”. Many pieces of research have studied the usefulness of eyes and their properties in various applications such as human-computer interaction, visual attention model assessment, detecting scene categories, and understanding the human state of the mind. Probably, the most influential study in this context is the seminal work of Yarbus [1] on the recognition of observer’s task from eye movements. He demonstrated that the viewers’ patterns of eye movements vary under different assigned tasks. His efforts emerged as the premise of the possibility of task decoding from eye movements which has the support of recent behavioral studies. For instance, Henderson *et al*. [2] studied the possibility of decoding four tasks of scene memorization, search, text reading and pseudo-text reading using multivariate pattern classification. Later, Borji and Itti [3] probed several experimental factors to assess the informativeness of eye movements for decoding the observer’s task. They utilized machine learning techniques to demonstrate a moderate, yet above chance, possibility of task recognition using eye movements. The observer’s task in their experiments is answering the original seven questions of Yarbus’s experiment. It includes answering questions in regard to free examination, identification of wealth, age estimation, activity prediction, remembering people, cloth, & objects, and estimating time between visits by watching a series of images that contain interaction between family members.

In the domain of technology, computer vision scientists adopted eye movements for human-computer interaction purposes. Bulling *et al*. [4] applied the eye movements in order to perform activity recognition. They exploited fixations, saccades and blinks to define a feature set, which is reduced via minimum redundancy maximum relevance (mRMR), and learned a support vector machine to determine any of the tasks of copying a text, reading a printed paper, taking handwritten notes, watching a video, and browsing the Web. In the context of multimedia analysis, Subramanian *et al*. [5] suggested the use of eye movements as sort of meta-tag information to discriminate interactive scenes, which depict social interactions, against non-interactive ones. Therefore, they clustered the fixations of several observers and computed their relation to form an interaction matrix, which represents the amounts of saccades from one cluster to the other one. They also extended the study to analyze the power of eye movements in distinguishing highly-expressive faces from mildly-expressive ones and recognition of nude content. Eventually, they concluded that eye movements provide a powerful tool in the semantic content analysis of a scene. Predicting the memorability of images is another application which may benefit from eye movements. Traditionally image content is analyzed in order to assess image memorability, e.g. [6], however, Mancas and Le Meur [7] applied eye movements and demonstrated the usefulness of eye movements and visual attention techniques for such a task. They indicated that there is a significant difference between the fixation duration of the top 20 most memorable and the bottom 20 less memorable images. They also established a link between inter-observer visual congruency [8] and the memorability of images.

The valence of a visual scene defines the extent to which a person is attracted (positive valence/pleasant) or repelled (negative valence/unpleasant) by the visual content. For example, everyone may have had the experience of the negative feelings caused by the threatening and frightening imagery and/or the pleasant feelings induced from watching colorful comforting pictures. Multimedia analysis programs such as video genre classification and content based image retrieval can benefit from valence recognition by identifying the amount of pleasantness of an image. The recognition of scene valence from eye movements is potentially possible. Nummenmaa *et al*. [9] demonstrated that eye movement patterns capture emotional content. They showed two images and asked observers to assess if the images are emotionally different while recording their eye movements. Afterwards, they measured four variables including first fixation latency, first fixation probability, gaze duration (total time looking on an image before shifting to the other image), and the number of first-pass fixations. Eventually, by analyzing the obtained data, they concluded that eye movement is affected while observing emotional content resulting in higher frequency of fixation and longer gaze duration on emotional stimulus compared to the neutral ones. Humphrey *et al*. [10] probed the ability of the rapid detection of emotive stimuli by investigating the performance of spotting such a stimuli in scenes which include highly salient regions. The conclusion is that while visual saliency influences eye movements, the effect is reliably reduced in the presence of emotional objects such that the initial fixations are more likely to lie on emotional objects than neutral visual salient ones. Similarly, Niu *et al*. [11] compared visual salience models against emotional stimuli and visual stimuli in order to understand their effect on gaze allocation. Their study reveals the inefficiency of visual salience models in predicting fixations on emotive regions. Thus, they conclude that emotional salience can override visual salience and alter the eye movements patterns.

In order to recognize image valence, Tavakoli *et al*. [12] employed eye movement based features. They extracted several features from human eye movement, particularly fixations and saccades, to recognize the valence of an image. They demonstrated that eye movement based features provide a better solution compared to the low-level visual descriptors. Although they demonstrate the overall usefulness of eye movements, their study lacks an in-depth analysis of the eye movement features. To compensate, in this paper, we analyze the eye movement features in the recognition of the valence of images. Several features are obtained from human eye movement data and a systematic machine learning approach is applied to investigate their fusion in order to identify the most influential feature set. In summary, the contributions of this study for image valence categorization are: 1) we examine the usefulness of histogram representations for encoding features extracted from eye movement and establish a baseline, 2) we thoroughly study the combination of features, 3) we try to identify the most influential features extracted from eye movements.

In the rest of this paper, we initially introduce the stimuli and data. It is followed by an explanation on the features extracted from the eye movements. Afterwards, we briefly discuss the pattern classification approach including dimension reduction techniques, the classifier, and performance measure. Finally, we report the experiments which are followed by a discussion.

## Data and Features

In this section, we initially explain the stimuli and the recorded eye movements. Afterwards, we discuss the properties of eye movements and describe the feature extraction procedure. Moreover, we spell out the motivation for each feature.

### Stimuli and Eye Movements

This study relies on a set of affective pictures and the recorded eye movements of people who observed them. The best set of affective images, known in psychological studies, is provided by *International Affective Picture System* (IAPS) [13]. It is a corpus of 1003 images with emotional rating which is reported in terms of means and variance of users’ Self-Assessment Manikin (SAM) scores. NUSEF dataset [14] provides the eye movements for a subset consisting of 287 affective images of the IAPS corpus, which are used in this study.

Initially, we analyzed the emotional information in terms of visual categories/classes using the affective information provided by IAPS. Table 1 reports the results for 12 different visual classes that one can identify in the set of images. As depicted, there is an emotional bias in different class categories probably because of the lack of enough images (e.g. building, animals) and the built-in category emotional bias (e.g. baby, rotten). Such a phenomena in combination with the role of fixation locations in the recognition of visual categories [15, 16] makes the study vulnerable to bias encoding, i.e. getting advantage of bias, which makes the fair analysis of eye movement factor difficult. To reduce the effect of the bias imposed by variation in visual categories (scenes), we focus on only one visual class, denoted as People & Daily Activity, which includes images of people involved in doing some activity.

**Table 1:** Emotional images and their visual classes.

Image Category	Number of Images			
	unpleasant	pleasant	neutral	all
Building	0	0	2	2
Food	0	2	2	4
Baby	0	8	0	8
Rotten	8	0	0	8
Abstract & Conceptual	3	0	6	9
Animals	0	11	6	17
Wild Animals	8	2	7	17
Nature	4	15	7	26
Objects	3	0	24	27
Nude & Porn	0	9	19	28
Activity	6	7	19	32
People & Daily Activity	29	24	56	109
Total	61	78	148	287

The following valence category is obtained by the emotional mean valence values reported by IAPS.

**Fig 1:**
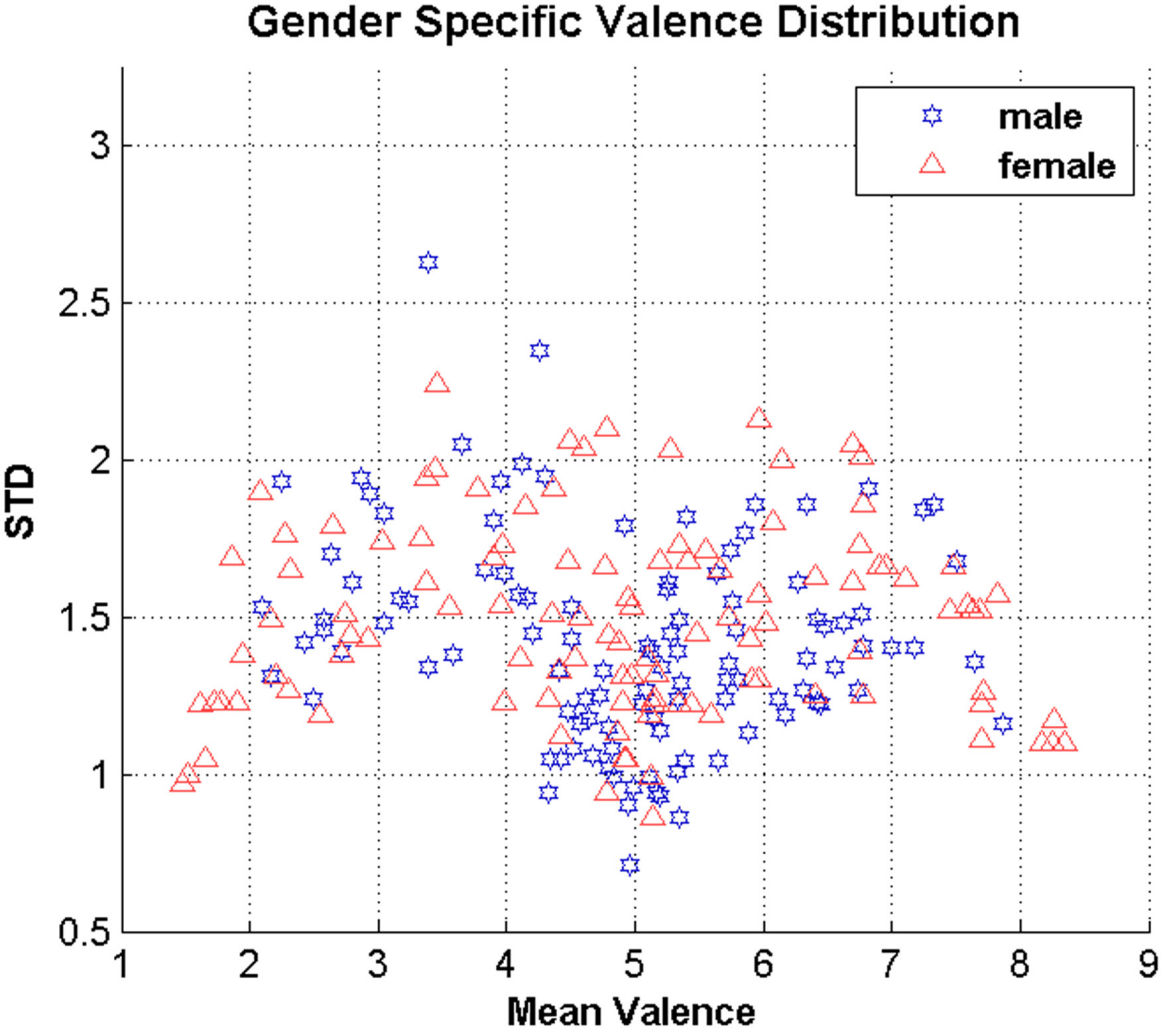
Analysis of gender and emotional distribution of images. As visualized, there is a difference between the ratings of female and male observers. Each image is visualized in terms of its mean valence and standard deviation ratings in regard to the genders.

The differences between the emotional behavior of female and male [17, 18] impose another concern in processing the eye movements for valence recognition. There are studies indicating the existence of gender differences in eye movement, e.g., one can infer personal traits and gender using eye movements [19]. Thus, the correlation of gender with eye movements and emotion is tried to be minimized in order to simplify the analysis of emotions using eye movements. In the first step, the 109 images of People & Daily Activity are studied for gender specific variations in terms of gender responsiveness. Fig 1 represents the mean valence and standard deviation of each image in terms of genders. It seems females are having more diverse responses, as their responses cover the whole span of valence. It is, however, very difficult to make the same conclusion as [18] about female responsiveness because of the limited amount of images.

**Fig 2:**
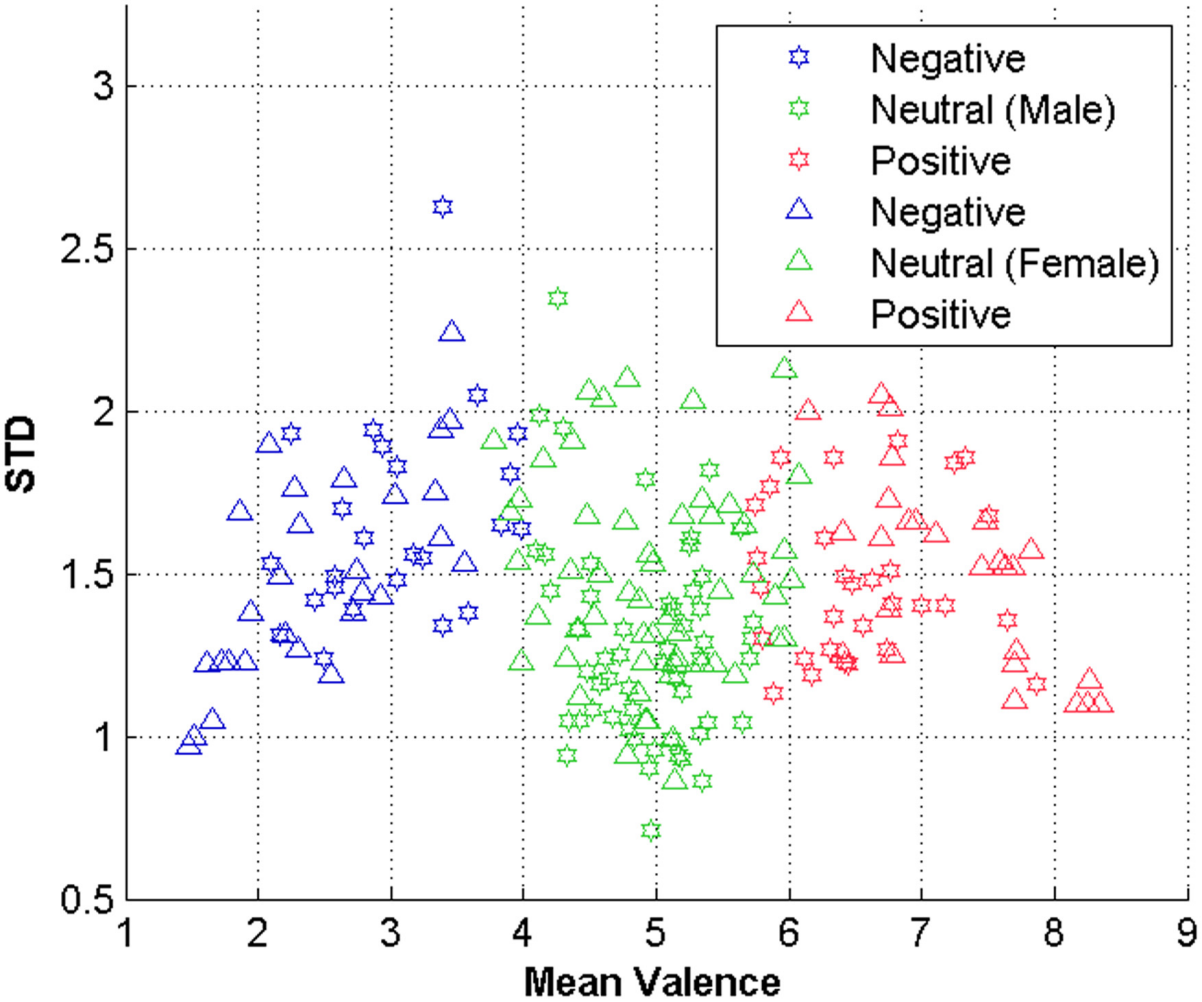
Valence categories and genders. To show the existence of differences between male and female genders, a fuzzy c-means is run to categorize the images based on their mean valence and standard deviation into three classes of unpleasant, neutral, and pleasant. Comparing gender specific results, the disagreement of genders on valence is evident.

**Fig 3:**
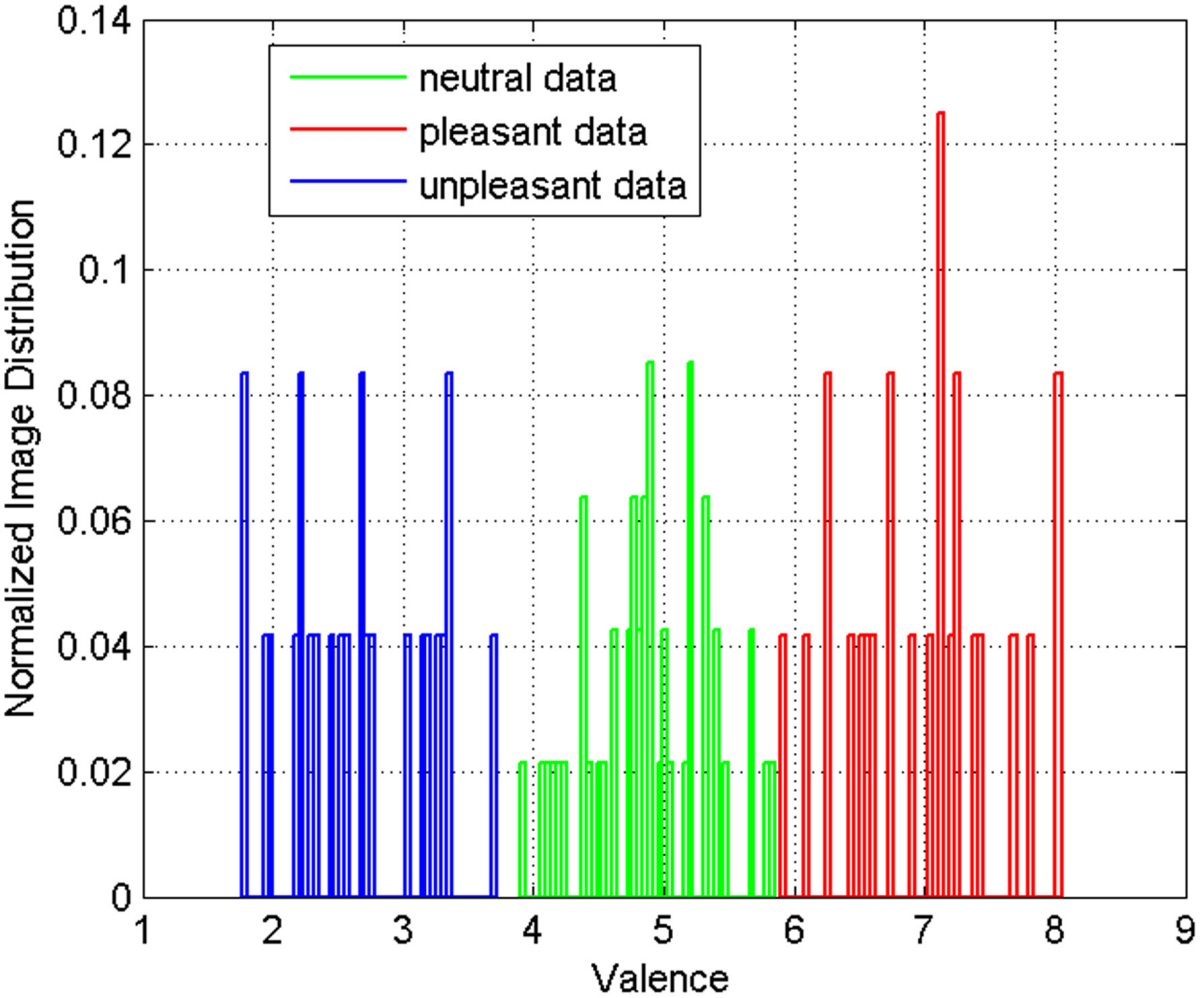
Distribution of images across the valence range in each class of unpleasant, neutral, and pleasant.

Furthermore, the effect of gender specific emotion on the three categories of unpleasant, pleasant, and neutral are studied. To obtain gender specific class categories for each image, we applied a Fuzzy c-means algorithm on male and female ratings. Fig 2 depicts the results in which one can observe that there are differences between the clusters taking genders into account. This is somewhat supported by the study of Bradley *et al*. [17] on sex differences in picture processing. Thus, we left out the images for which the valence is strongly disagreed by human annotators between genders to avoid gender-specific bias. The final image set consists of 95 images, which includes 47 neutral, 24 pleasant, and 24 unpleasant images. Table 2 reports the name of these images and their emotional category. The emotional labels are obtained by assigning the images with mean valence in the range of 3.8–5.8 to neutral and every image with a lower mean valence to unpleasant and those with mean valence above 5.8 are considered as pleasant. Fig 3 represents the span of the valence for the final image set in each emotional category. As depicted, the final set of images covers the almost the whole span of valence values.

**Table 2:** Images used in the study.

Valence	Image ID
Neutral	‘2020’, ‘2102’, ‘2104’, ‘2130’, ‘2190’, ‘2200’, ‘2271’, ‘2272’, ‘2280’, ‘2210’, ‘2214’, ‘2215’, ‘2220’, ‘2221’, ‘2230’, ‘2305’, ‘2357’, ‘2372’, ‘2383’, ‘2385’, ‘2393’, ‘2396’, ‘2397’, ‘2435’, ‘2441’, ‘2485’, ‘2487’, ‘2491’, ‘2493’, ‘2495’, ‘2499’, ‘2512’, ‘2513’, ‘2516’, ‘2520’, ‘2595’, ‘2635’, ‘2690’, ‘2704’, ‘2749’, ‘2770’, ‘2780’, ‘2795’, ‘2830’, ‘2840’, ‘2870’, ‘7506’
Pleasant	‘1340’, ‘1999’, ‘2000’, ‘2010’, ‘2037’, ‘2091’, ‘2092’, ‘2154’, ‘2222’, ‘2304’, ‘2339’, ‘2340’, ‘2341’, ‘2358’, ‘2362’, ‘2391’, ‘2501’, ‘2530’, ‘2620’, ‘2650’, ‘4617’, ‘5410’, ‘7325’, ‘8497’
Unpleasant	‘2095’, ‘2110’, ‘2120’, ‘2141’, ‘2205’, ‘2276’, ‘2278’, ‘2490’, ‘2590’, ‘2691’, ‘2710’, ‘2750’, ‘3500’, ‘3530’, ‘4621’, ‘6243’, ‘6313’, ‘6315’, ‘6360’, ‘6370’, ‘6530’, ‘6550’, ‘6560’, ‘6561’

The 95 images from IAPS used in this study and their valence category.

The eye movements for the images are provided by the NUSEF dataset. As reported by [14], the dataset consists of the eye movements of, on average, 25 subjects (observers) per image. The observers are within the age of 18–35 with the mean range of 24.9. The images are all normalized to the size of 1024 × 768. Each image was shown on a 17 inch LCD monitor at the distance of 3 feet for 5 seconds followed by a 2-second presentation of a gray mask in a free viewing task. The eye movements have been recorded at the sampling rate of 30Hz using an ASL eye tracker which is within a 1° accuracy (error radius of approximately 5 pixels on screen) for the configuration upon a 9-point gaze calibration. A fixation point represents the screen location in which the gaze remains within 2° visual angle for at least 100 milliseconds, which results in the total fixations of 29904 for the 95 images of this study. An advantage of the eye movements provided by NUSEF is that the emotion-evoking images are presented randomly along with around 500 images of non-emotive (less-emotive) content. Thus, one can expect that the inter-effect of emotions evoked by IAPS images can be less than usual compared with the case of continues eye movement recording using only IAPS images.

### Features

The eye movement data consist of an ordered set of fixations and saccades. A fixation is defined by a quadruple *F* = (*x*, *y*, *t*_*start*_, *t*_*end*_) containing image location coordinates (*x*, *y*) and time stamps indicating when the gaze arrived (*t*_*start*_) and left (*t*_*end*_) this location. A saccade is defined as *S* = (*x*_*start*_, *y*_*start*_, *x*_*end*_, *y*_*end*_, *t*_*start*_, *t*_*end*_) denoting the locations and time stamps for the start and end of the saccade. The proposed features use both fixation and saccade information.

The fixation location can be interpreted as an indication of cognitive process in the mind, e.g., gaze location can be used for identifying observers tasks [3]. Furthermore, general findings have been that negative affects produce attentional narrowing and positive affects broaden the attentional focus [20, 21]. In other words, one shall expect that the fixation density is more compact for exposure to negative stimuli compared with the positive ones. Thus, *fixation location*—FL(*F*_*i*_) = (*x*_*i*_, *y*_*i*_)—as the representative of the observers focus of attention is expected to be a useful property for valence recognition.

Along with the fixation location, *fixation duration*—FD(*F*_*i*_) = *t*_*end*, *i*_ − *t*_*start*, *i*_—characteristic is a well-studied feature. Assuming the fixation location corresponds to the spatial locus of cognitive process, one can hypothesize that the fixation duration indicates the duration of the cognitive process. Consequently, the combination of fixation duration and fixation location are also believed to be dependent variables conveying the cognitive state of the mind, particularly in complex cognitive processes such as reading [22, 23]. Nonetheless, the fixation duration is suggested to change during affective scene viewing. Studies have indicated that fixation duration is correlated with the emotional content, threat-based affective responses and anxiety [20]. Particularly, the decrease in positive affect correlates significantly with fixation duration [24].

The exposure to affective stimulus also affects saccadic behavior such as saccade length, saccade velocity, and saccade angle change. The *saccade length* captures the distance between fixation points and defines the length of the shifts in the person’s gaze, i.e., $\text{SL}(S_{i})=\|{\vec{S}}_{i}\|$. It is demonstrated that saccade lengths behavior is strongly different during viewing negative or threat-based stimuli than during pleasant and neutral images. It is demonstrated that the saccade length is longer for unpleasant stimuli compared to pleasant stimuli in the presence of emotionally high-arousal content [25].

Simola et al. [25] showed that emotional content alters the angular behavior of eye movements meanwhile studying the interaction of valence and emotional arousal factors by measuring the saccade angle. The saccade angle indicates to what direction the gaze is moving. Considering the saccades happening after the first fixation on emotionally rich regions, the saccade angle is larger for unpleasant stimuli compared with pleasant stimuli when the emotional arousal level is low (It is smaller for high-arousal unpleasant stimuli.) [25]. We encoded the angular information using two features, the *saccade slope* (SS) and *saccade orientation* (SO). The saccade slope is the direction and steepness of the straight line that connects two successive eye locations, i.e., SS(*S*_*i*_) = (*y*_*end*, *i*_ − *y*_*start*, *i*_)/(*x*_*end*, *i*_ − *x*_*start*, *i*_). We approximated the slope by computing the slope of the line connecting two succeeding fixations. It reveals if there has been a vertical movement that resulted in a fixation or a horizontal one. Saccade orientation, SO(*S*_*i*_, *S*_*i*−1_), is the angle between two succeeding saccades. It is computed in terms of $\cos^{-1}\left(\left({\vec{S}}_{i}\cdot {\vec{S}}_{i-1}\right)/(∥{\vec{S}}_{i}∥\cdot ∥{\vec{S}}_{i-1}∥)\right)$. The saccade orientation describes the behavior of one saccade taking its predecessor into account. In fact, it complements saccade slope and facilitates inference about the angular behavior of saccades.

*Saccade velocity* (SV) tells how fast the gaze is moving by expressing the rate of change in eye locations, i.e., $\text{SV(}S_{i})=∥{\vec{S}}_{i}∥/\left(t_{end,i}-t_{start,i}\right)$. While too slow or too fast saccade velocities can be the indication of syndromes or diseases, e.g. cerebellar syndrome and opsoclonus syndrome, the change in saccade velocities help decoding observers’ emotional state. The saccade velocity is proven to change with respect to the emotional content encounters [26]. Furthermore, examining velocity and gaze direction under anti-saccadic and pro-saccadic conditions in the presence of emotional stimuli reveals dissimilar response times for neutral, unpleasant, and pleasant stimuli [21, 27]. The saccade velocity is often useful in the recognition of anxiety [28] which is linked to the exposure to the negative stimulus [29]. Thus, features based on saccade velocity are expected to contribute to the recognition of valence. The saccade velocity computation relies on saccade length and *saccade duration*, SD(*S*_*i*_) = *t*_*end*, *i*_ − *t*_*start*, *i*_. Saccade duration is the time it takes to move the eye from one location to the next one. Along with the role of saccade duration in the computation of the velocity, there are also cases of links between cognitive processes during saccades [30]. Thus, we speculate possible contribution of saccade duration and adopt it as a complementary feature with saccade velocity.

#### Feature representation

**Fig 4:**
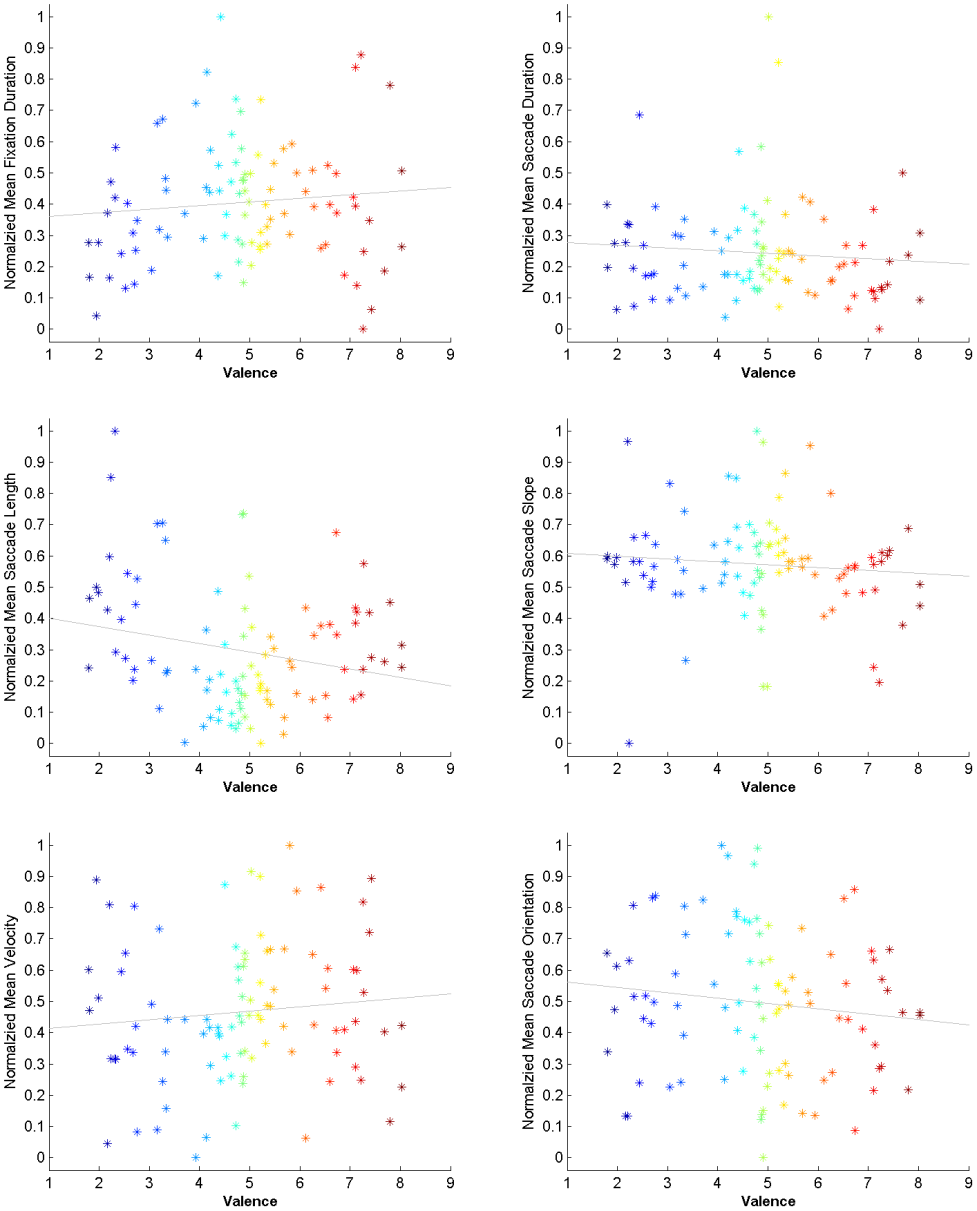
Mean value features versus valence. From left to right: mean fixation duration (m = 0.012, p = 1.32e-36), mean saccade duration (m = -0.008, p = 1.11e-26), mean saccade length (m = -0.027, p = 1.77e-25), mean saccade slope (m = -0.002, p = 1.49e-35), mean saccade velocity(m = 0.004, p = 0.14), mean saccade orientation (m = -0.017, p = 5.19e-37).

Traditionally, behavioral studies often consider the mean value of the above features in order to provide supporting evidence for a hypothesis. To assess the usefulness of feature representation using mean values, Fig 4 visualizes the normalized mean value of some of these properties versus the valence of the images and the corresponding regression line. For each image, the normalized mean value is obtained by normalizing the average value of the features (including fixation duration, saccade duration, saccade velocity, saccade length, saccade slope and orientation) across all observers to the range of [0, 1]. The regression analysis shows that the mean of some of the features are meaningful in regard to valence; these features are: fixation duration (p = 1.32e-36), saccade duration (p = 1.11e-26), saccade length (p = 1.77e-25), saccade orientation (p = 5.19e-37), saccade slope (p = 1.49e-35). However, the mean of saccade velocity (p = 0.14 > 0.05) is not promising. We further performed a 1-way ANOVA analysis with the features as factors indicating the possibility of interaction between at least two of the features (F = 134, P = 1.72e-93). We complemented the test with a series of pairwise post hoc tests consisting of Least Significant Difference(LSD) and Tukey-Kramer. The results show that, except the pair of fixation duration and saccade orientation features, all the feature pairs have significant interactions (p < 0.05).

Although it is possible to use the mean values as features, such simple features do not necessarily suffice to decode complex tasks [3], e.g., Kanan *et al*. [31] and Greene *et al*. [32] fail to decode observers’ task using mean fixation duration with RBF & Linear SVM, respectively. Alternatively, we adopted a histogram representation which provides a feature distribution across observers. This provides a probabilistic interpretation and improves task identification. The features belong to two main feature category based on fixations and saccades. Fixation based features utilize fixation location to compute fixation density map a.k.a saliency map (In this study, the fixation density map, i.e., smoothed fixation map, represents the saliency map so the two terms are used interchangeably.), saliency histogram, fixation histogram, top ten salient locations and also the duration in terms of a histogram of fixation duration. Saccade based features are histograms of saccade slope, saccade length, saccade velocity, saccade orientation, and saccade duration.

The saliency map is a smoothed fixation map by a Gaussian kernel of *σ* = 10 which produces a compact and accurate representation of fixation density and normalizes the map to [0, 1]. The choice of *σ* is such that it corresponds to almost 2° visual angle equal to the amount used for the area corresponding to fixation in NUSEF database during fixation detection. Furthermore, the size of saliency map is reduced to 15 × 20 and is vectorized to make a feature vector. Based on comparison with the original map, the down-sampled saliency map gives more weight to approximately the 40% most salient regions of the map which is reported to be equivalent to around 95% of human performance [33]. The salience histogram is a histogram of saliency values at the fixations on the saliency map and consists of 10 equally distributed bins. The salience histogram partially represents the discrepancy of observers viewing path. The salience histogram can be complemented with the fixation histogram which provides spatial information about the attended contents. Fixation histogram encodes the number of fixation occurrence in the image regions; this study considers 256 regions obtained from a 16 × 16 grid. The selection of 16 × 16 grid provides an image patch size of 48 × 64, which is approximately 10 times the size of possible eye tracker spatial error. In fact, we can slightly capture the amount of sparsity in fixation data with respect to visited regions (patches). Top ten salient locations represents the coordinates of the 10 strongest local maxima in the saliency map which are extracted using the inhibition of return mechanism [34]. It conveys the spatial information about the most agreed locations of the scene which is demonstrated of having a contribution in attention allocation depending on the stimulus characteristic [35]. The fixation duration information is encoded in terms of a histogram which represents the distribution of duration for the free-viewing task. It is obtained by quantizing the range of minimum and maximum duration time into 60 uniformly distributed bins. The number of bins is chosen in relation to time quantiles.

The saccade based features are all histogram based representations of the aforementioned saccade features. The histogram representation reflects the mean—similar to traditional behavioral studies e.g. [29]—meanwhile carrying more information by providing the probability distribution of the occurrence of the values of a property. For each saccade property, the corresponding histogram is built by choosing the centers of bins such that they are uniformly distributed between the minimum and maximum values of the corresponding property. The histogram of saccade slope consists of 30 bins in which the selection of bins considers a quantization of 6° for the slope values in the range 0° to 180°. We chose 50 bins to represent the histogram of saccade length because it results in a less sparse histogram, similarly, the histogram of saccade velocity consists of 50 bins. The histogram of saccade orientation uses 36 bins to provide a 10° quantization for the range of orientations. Similar to the histogram of fixation duration, the histogram of saccade duration consists of 60 bins. Eventually, the concatenation of all the extracted features results in a feature vector of 872.

## Pattern Classification

In this section, we discuss the pattern classification basis of the experiments. While Tavakoli *et al*. [12] framework applied a late fusion scheme in which a classifier is trained per-feature, we adopt an early fusion mechanism in this paper. Thus, the framework consists of feature extraction, explained above, feature reduction/selection and classification. Initially, we will discuss the necessity of feature reduction/selection. Afterwards, we explain the classification protocol and the performance metric.

The increase in the dimensionality of the feature vector can result in the fast growth of the volume of the space that makes the available data become somewhat sparse. Therefore, having a fixed number of training images, the predictive power reduces as the dimensionality increases. To deal with this scenario, we applied series of feature reduction/selection schemes combined with a SVM classifier in order to avoid such a phenomena since the feature vector of dimension 872 is much larger than the total number of 95 images. Moreover, the application of several dimension reduction techniques (including reduction-based and decomposition-based methods) helps to identify the best contributing features. This approach is fairly common in image processing, data mining, and machine learning domains because depending on the features one specific feature selection may necessarily not perform well, e.g., [36]. We applied several feature selection schemes such as evolutionary-based decomposition and feature selection/fusion methods by sub-selecting components with over 90% contribution to the variance. We also tested Sequential Forward Selection (SFS) and Sequential Backward Selection (SBS) [37] along with Principal Component Analysis (PCA), and Singular Value Decomposition (SVD) as a basis. Please check S1 File for the details on feature selection techniques along with [38] for an in-depth review of evolutionary methods.

We utilized Support Vector Machine (SVM) to classify the images into three classes of unpleasant, neutral, and pleasant. In general, SVM uses a hyperplane or a set of hyperplanes to discriminate two or more classes of the data from each other by maximizing the distance between the hyperplane and the closest points of each class while the classification error is minimized [39, 40]. It has a sound theoretical foundation and generates global solutions without getting stuck in a local minima [41]. We applied three SVM kernels of linear, polynomial, and radial basis function (RBF). We considered three kernels because the stochastic nature of evolutionary methods for feature reduction/selection makes clear pre-understanding about the data separability difficult. The choice of kernels was motivated by the fact that linear kernel is expected to perform better for linearly separable data while RBF and Polynomial kernels perform better for non-linearly separable data points which require to be mapped to a different dimension within which they are separable. The classification scheme is 1-versus-rest where there exists one classifier per valence category. The generalization error is estimated using repeated cross-validation (CV), resulting in 10 repetitions of a 10 fold CV with three sets of train, validation, and test of the ratios of 0.9, 0.05, 0.05, respectively. In each fold, we guarantee an equal number of samples from each class category following a conservative sampling strategy [42, 43].

The most common and popular metric for evaluation of classifiers is confusion matrix which describes the classification performance details per-class category. We must, however, summarize the confusion matrix into one score in order to perform a wrapper based feature selection. A confusion matrix can be summarized in terms of a single measure of performance like accuracy, F-measure, precision, and recall. Nonetheless, such single measures are influenced by the class distribution and sample bias. To address the mentioned problems, [44] introduced *Bookmaker Informedness* by taking the difference between the correct/incorrect informed decisions and uninformed (random) choices into account. The *Bookmaker Informedness* provides a measure between -1 and +1 in which +1 represents perfectly correct performance, -1 indicates the perversely incorrect response and 0 is the chance level. Therefore, we adopted *Bookmaker Informedness* as a normalized and unbiased performance metric to facilitate automatic assessment during feature fusion and comparison. The mean accuracy is also provided along the informedness and the confusion matrices are available in S1 File. We analyzed all the classification results using an n-way ANOVA in the relevant points.

## Experiments

In the experiments, we evaluate the features and their contribution in regard to several conditions. Initially, we evaluate each individual feature to establish a base-line. We test both histogram representation and mean-value over observation period representation. We then run several dimension reduction procedures using conventional and evolutionary-based approaches and try to find the maximum possible performance and identify the most contributing features.

### Experiment I: Baseline

**Fig 5:**
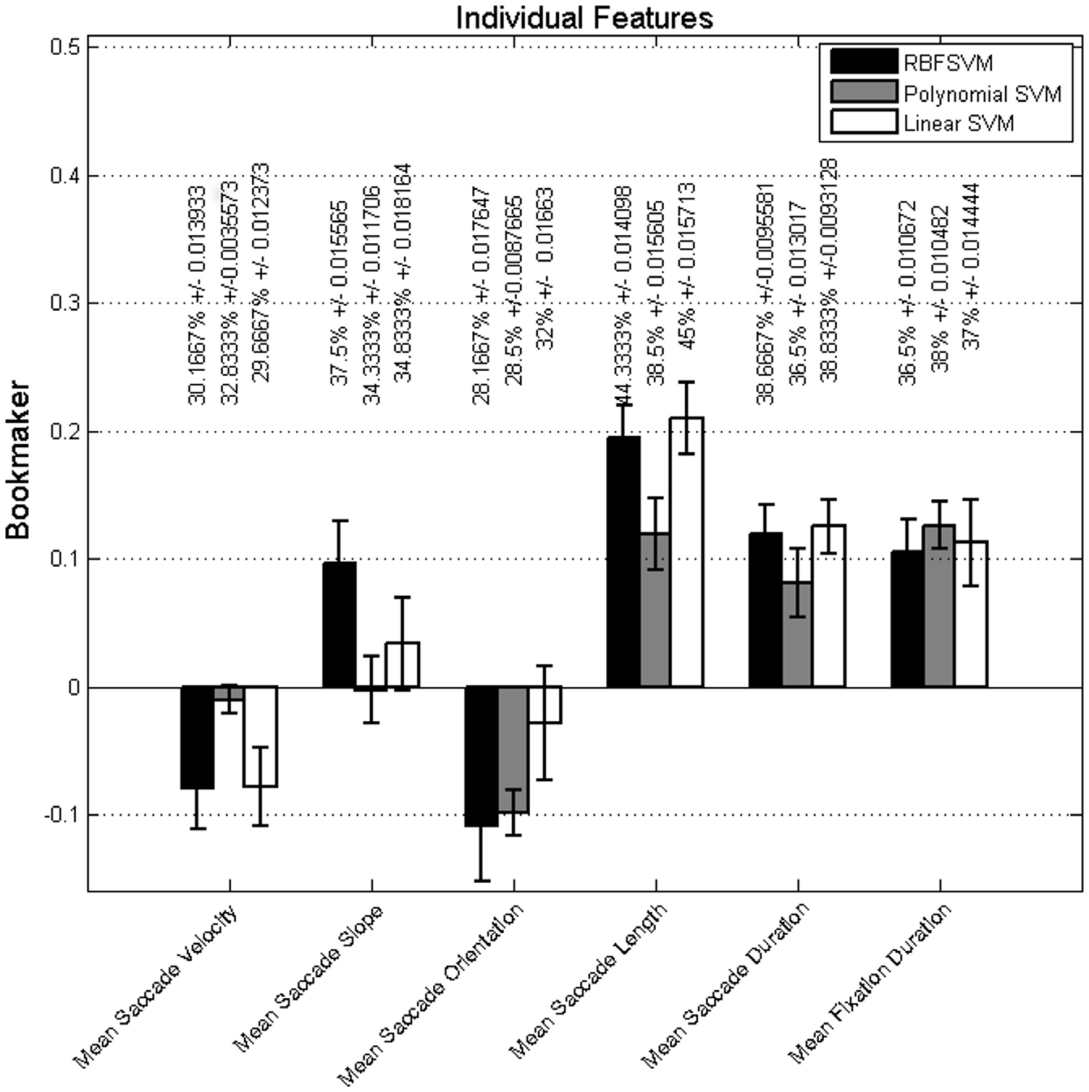
Baseline mean performance for mean value features in terms of bookmaker for classification of images into three class of unpleasant, neutral, and pleasant. The mean of classification accuracy across folds and repetitions (%) and their associated standard errors are also added to each bar as a second measurement unit.

To establish a baseline, we investigated the features individually and all together. We assessed two feature representations in a three-class classification to identify unpleasant, neutral and pleasant images from the extracted features. The performance of traditional features (mean-value based features) are summarized in Fig 5. As depicted, mean saccade length performs better than any other feature. Also, mean saccade duration, mean fixation duration, and mean saccade slope are performing above chance. The mean saccade velocity and mean saccade orientation are not discriminating enough.

**Fig 6:**
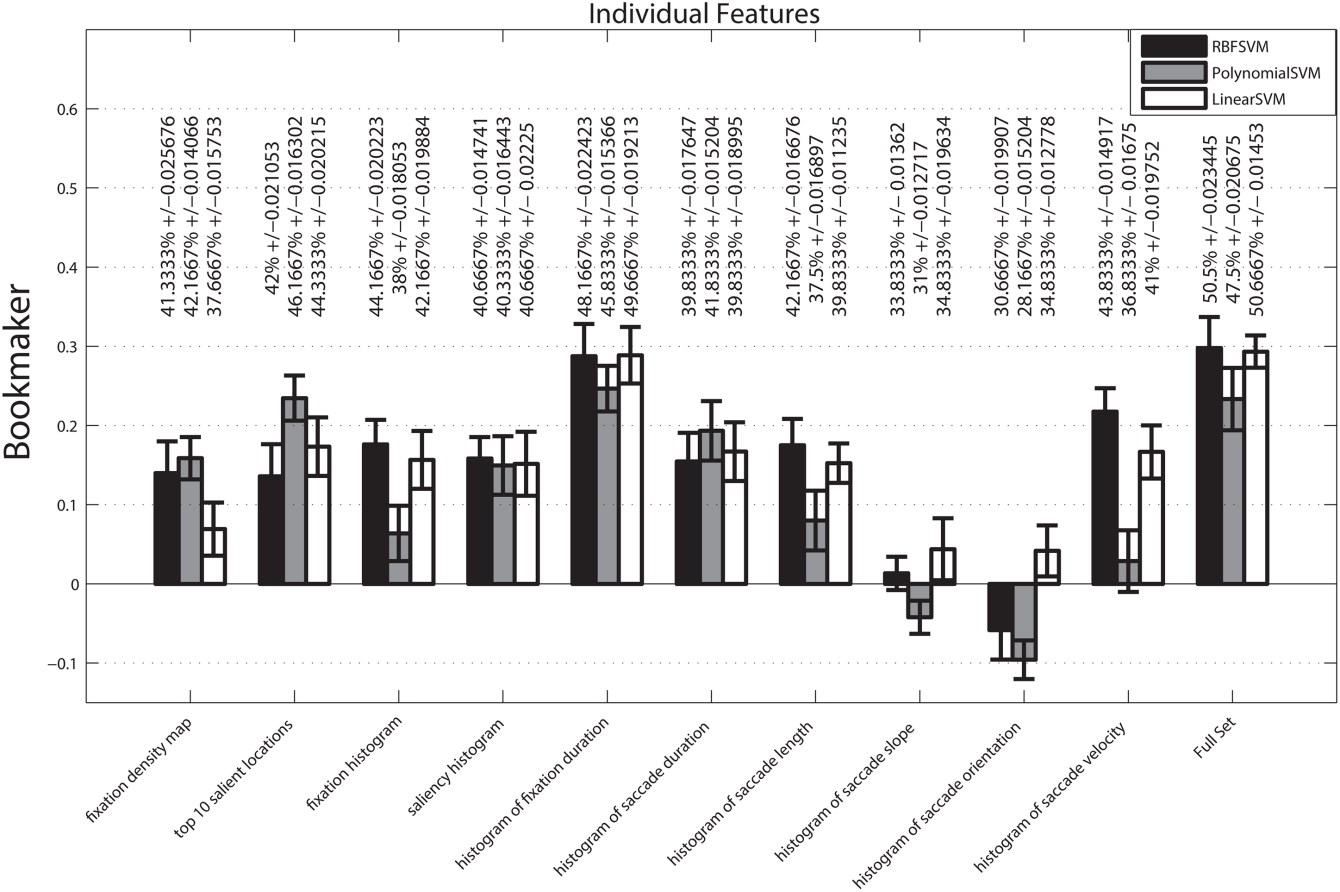
Baseline performance of individual histogram-based features in terms of bookmaker for classification of images into three class of unpleasant, neutral, and pleasant. The mean of classification accuracy across folds and repetitions (%) and their associated standard errors are added to each bar as a second measurement unit.

We performed a similar experiment using the histogram-based representation of the features and fixation location based features. The result is summarized in Fig 6. The experiment reveals that histogram of fixation duration and the full set (fusion of all the features) have convincing classification performances among the assessed feature types with the full set having a slight advantage over the fixation duration. The saccade slope and saccade orientation have the lowest overall performance and, depending on the SVM kernel, they even sometimes perform worse than chance. The statistical analysis of the results indicates that there is a significant statistical difference among features (p = 0 < 0.05), classifiers (p = 0.0038 < 0.05), saccade-based features vs fixation-based features (p = 0 < 0.05), and the interactions of features and classifiers (p = 0.0024 < 0.05). The interaction of saccade-based features and classifiers vs fixation-based features and classifiers, however, shows no significant statistical difference (p = 0.07 > 0.05). Further analysis reveals that there is a statistical significant difference between fixation-based, saccade-based features and their combination. A closer analysis of the feature performances reveals that saliency map, top 10 salient locations, fixation histogram, saliency histogram, saccade duration, saccade length, and saccade velocity are significantly different from saccade slope, saccade orientation, fixation duration, and mixture of all features. The mixture of all features shows no significant difference only with fixation duration. The saccade slope only shows the lack of significance compared with saccade orientation.

Comparing the results of mean-value based features and the corresponding histogram-based features in terms of bookmaker reveals that histogram representation improves the performance of fixation duration and saccade duration significantly. Histogram representation does not improve the performance of saccade slope, saccade orientation, and saccade length. The saccade velocity is a peculiar feature in this comparison because the histogram representations reveal the usefulness of a feature which its mean value does not work better than chance in the assigned task. Taking the improvements by histogram-representation into consideration, we focused on these features on the rest of the experiments.

### Experiment II: Feature Combination

In this experiment, we focused on the feasibility of performance improvement using dimension reduction with conventional and evolutionary based approaches. We also identified the most contributing features in the task of image valence recognition where three class of unpleasant, neutral, and pleasant exist.

We used both conventional decomposition approaches and evolutionary techniques. In the first category, we adopted PCA and SFS as main approach and SVD & SBS as alternative decomposition approaches. Among evolutionary methods, we used variations of binary genetic algorithm (GA) and a modified version of particle swarm optimization (PSO). We used both GA decomposition (which identifies a subset of feature points) and GA early fusion approach (which identifies a subset of feature types). We only reported the best performing techniques. Please check S1 File for more information on parameter settings, methods, and extra results.

**Fig 7:**
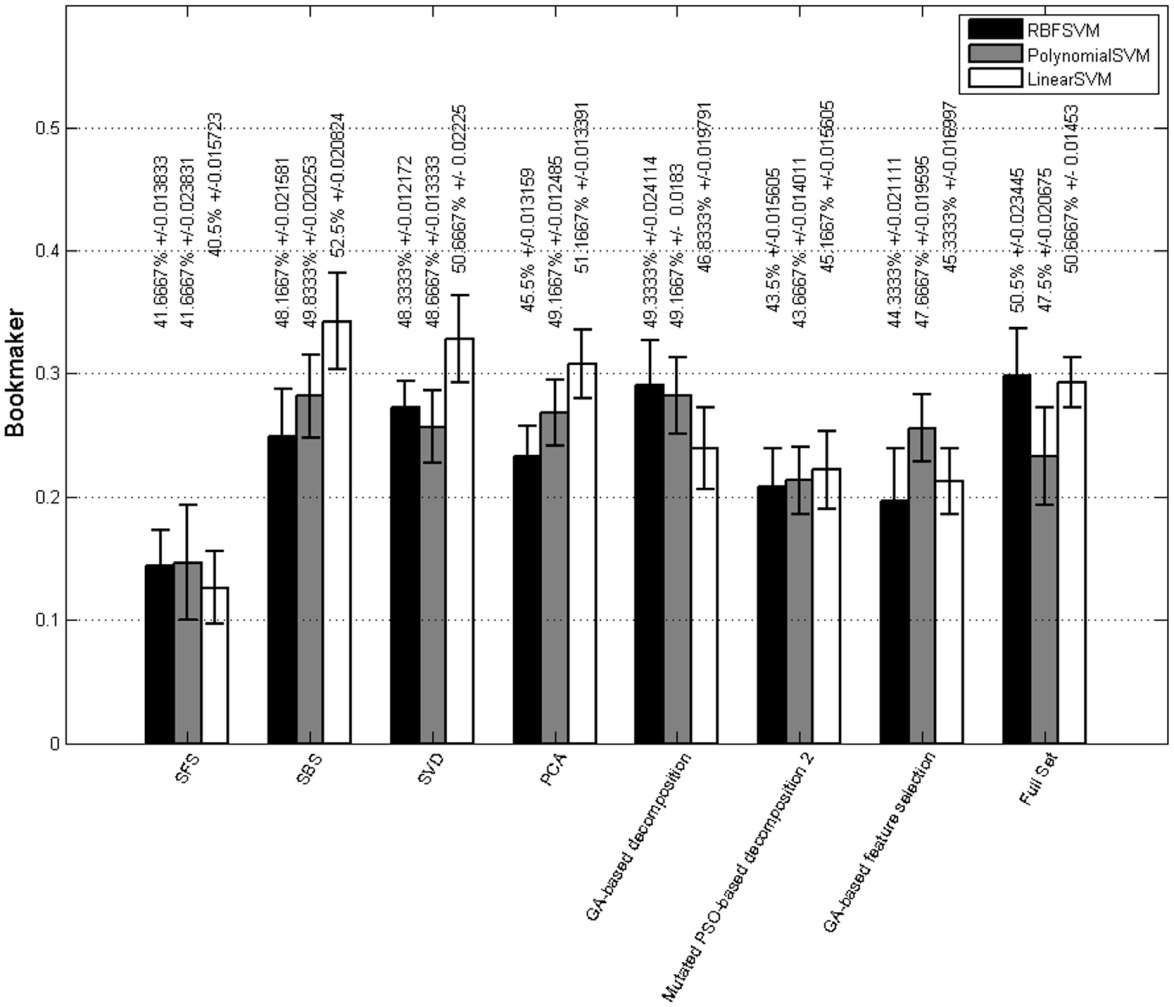
Performance of conventional and evolutionary based decomposition methods. Mean of classification accuracy across folds and repetitions (%) and their associated standard errors are added to each bar as a second measurement unit.

The classification results using various dimension reduction techniques are demonstrated in Fig 7. The overall advantage of the combination of features using SBS and linear SVM is absolutely evident from the results. The SFS approach shows considerably lower performance compared to all the feature reduction methods. SBS and linear SVM achieves the highest performance followed by SVD and PCA. Considering GA based techniques, we observed that both GA decomposition and fusion perform above chance level. The GA decomposition and RBF SVM achieve a performance close to the full set. The PSO-based decomposition performs above chance level consistently across all kernels. It is performing 0.1 above SFS and 0.08 below the full set in terms of bookmaker metric. We performed a statistical analysis and realized that there is no significant statistical difference among classifiers (p = 0.3717 > 0.05) and the interactions of approaches and classifiers (p = 0.5435 > 0.05). But, we observed a significant statistical difference among feature selections (p = 0 < 0.05). SFS has a significant statistical difference with *Mutated PSO-based decomposition 2* and *GA-based feature selection*. The SBS, SVD, PCA, and Full Set are significantly statistically different from the SFS method. The *Mutated PSO-based decomposition 2* and *GA-based feature selection* demonstrate no significant statistical difference with SBS, SVD, PCA, and full set.

The combination of SBS feature selection and linear SVM provides the best performance in terms of bookmaker and accuracy. Consequently, we examined the feature vector resulted from this procedure in order to identify the contributing features. The final feature vector is reduced by the ratio of 7.9 on average for the 100 runs (10 repetitions, 10 fold), i.e., the final feature vector using this method is almost of size 109. Interestingly, all the features have some degree of contribution with the average number of feature point contribution over 100 runs as follows: saliency map (0.58), top 10 salient locations (0.18), fixation histogram (0.14), histogram of fixation duration (0.07), saliency histogram (0.05), histogram of saccade duration (0.04), histogram of saccade length (0.02), histogram of saccade velocity (0.01), histogram of saccade slope (0.004), histogram of saccade orientation (0.002). As observed the fixation-location-based features have the most contribution, followed by the fixation duration and saccade based features. In order to make a conclusion about the most contributing features, we looked into the features selected by all the combination of feature selections and classifiers across all folds and repetitions of the cross-validation. Among the features, saliency map and fixation duration have the highest contribution among all the feature selection schemes with on average selection of 19% and 11% of their feature points, respectively. Furthermore, we learned that the mostly selected feature types are *‘saliency map’*, *‘histogram of saccade slope’*, *‘histogram of fixation duration’*, and *‘fixation histogram’*. The selected features highlight the influence of fixation information (fixation location and fixation duration) and angular behavior of eye movements in the recognition of the valence of images.

## Discussion and Conclusion

Successful image valence recognition results, demonstrated in this study, provide further evidence for the contribution of eye movements for the recognition of emotional valence of a scene. This partially supports the general findings of [10] and [11] which demonstrate eye movement is altered in the presence of emotional stimuli. Furthermore, the results also relatively confirm the effect of emotional stimuli on the observers’ eye movements in terms of several previously investigated features including saccade length [25], fixation duration [20, 24], fixation location [21]. It is worth noting that we could not observer contribution from saccade velocity [26] and saccade angle [25] unless adapting the histogram representation and taking the interaction of features into account. We also identified contribution from saccade duration which, to our knowledge, is not explicitly studied in relation to emotion. We demonstrated that it is possible to recognize the scene valence from observers’ eye movements akin to [12]. Furthermore, we investigated the interaction between features and identified the most contributing ones consisting of saliency map, fixation histogram, histogram of fixation duration, and histogram of saccade slope for the recognition of pleasant, unpleasant and neutral scenes. The selected features put more weight on fixation information (both location and duration) and angular saccade information.

Does the feature representation matter? There are usually two feature representations, 1) mean value of features (e.g., mean fixation duration) and 2) histogram of features (e.g., the histogram of fixation duration). The first one is often adopted in the behavioral studies and the latter is used in the technology domain related problems such as [12, 15]. We compared the two feature representations and the experiments revealed that both representations are useful for valence recognition. The histogram representation, however, outperforms the mean value representation significantly; for example, the histogram of fixation duration performs almost 3 times better than mean fixation duration. We also observed that saccade velocity performance improves from below chance to above chance by adapting histogram representation. Thus, we can argue that feature representation matters and the histogram representation is preferable in the valence recognition. This also provides some motivation for behavioral studies to investigate other possible feature representations.

How easy is scene valence inference from eye movements in uncontrolled conditions? What may affect the success? We have to assert that there is no concrete answer to this question because the parameter space is gigantic and there are a lot of factors involved. The stimulus is the first crucial factor since the correlation between visual content and emotional message of the scene can be complicated [11]. This influences the identification of contributing eye movements and there is no guarantee for observing similar feature interactions. Nonetheless, the visual content with a bias towards a specific emotion can cause difficulties for machine learning based studies. The observers are the second influential factor which introduce many subjective compounds to the problem. One example, which we identified and addressed, is the role of gender that affects the performance of the system. Moreover, the background of observers such as their clinical status [45] and cultural background [46] can affect the success of this kind of studies and applications.

Aside from the mentioned factors, there are technical factors that may affect the success of image valence decoding. The eye tracker accuracy, precision and sampling rate are crucial parameters as they affect the quality and quantity of the eye movements; which has an impact on the feature extraction, for instance, the approximation of the saccade length close to actual amount of eye movement is possible with the help of a high sampling rate tracker. The feature extraction & representation and the machine learning method also affect the result. There are also neglected aspects of the eye movement such as its temporal behavior which may introduce even more sophisticated feature extraction and representation schemes. Such schemes will introduce new issues such as the number of quantization step, normalization, sampling, and etc. The combination of all these parameters and factors makes decoding eye movement related tasks difficult. We avoided such a complication for feature extraction by choosing the quantization step based on intuition and the study of the underlying data to make sure the histograms are not very sparse. Despite we compared the performance of the adopted feature extraction with the simplest possible feature representation (mean value) to avoid arguments on their usefulness, it is still worth to investigate the trade-off between complicated feature representations and information gain (this goes beyond current study). A similar issue holds for the choice of classifier, a good exemplar is the Yarbus’s task decoding where two contradicting results are achieved using the exact same data using different classifiers (please refer to [32] and [3] for details).

The emphasis of the current research for the scene valence recognition is on the technological applications though may be interesting for behavioral studies by comparing the feature representations. Potential applications include multimedia analysis, human-computer interaction, and next level of technologies equipped with eye-trackers such as glasses, displays, and augmented reality helmets. Despite there is a long way in this respect and we may have to wait some years in order to have the necessary hardware and frameworks available, the successful results of the current study motivate further investigations to assess various perspectives in order to provide robust algorithms. One direction of the research can be the assessment of the usability of the off-the-shelf eye trackers like Tobii EyeX and EyeTribe controllers in valence recognition and observer task decoding. These controllers provide affordable eye tracking solutions suitable for human-computer interaction purposes. Investigating the temporal information obtainable from eye movements is yet another interesting research direction. Eventually, augmenting eye movements with other biological cues such as heart rate is an interesting research domain for studying various scenarios such as scene valence recognition and emotion analysis.

## Supporting Information

S1 File Supporting text, figures, and tables. Fig A in S1 File, dimension reduction mechanism. Fig B in S1 File, performance achieved by conventional and evolutionary based decomposition methods. Fig C in S1 File, analysis of individual features using bagging approaches. Fig D in S1 File, analysis of features combinations using bagging approaches. Table A in S1 File, the initial setup of PSO. Table B in S1 File, parameter settings of the variations of PSO approach employed in this study. Table C in S1 File, sample size and feature combination. Table D in S1 File, confusion matrices: mean features and RBF SVM. Table E in S1 File, confusion matrices: mean features and polynomial SVM. Table F in S1 File, confusion matrices: mean features and linear SVM. Table G in S1 File, confusion matrices: individual features and RBF SVM. Table H in S1 File, confusion matrices: individual features and polynomial SVM. Table I in S1 File, confusion matrices: individual features and linear SVM. Table J in S1 File, confusion matrices: feature selection/decomposition and RBF SVM. Table K in S1 File, confusion matrices: feature selection/decomposition and polynomial SVM. Table L in S1 File, confusion matrices: feature selection/decomposition and linear SVM.
(PDF)
