# Correction: Predicting the Valence of a Scene from Observers' Eye Movements

**DOI:** 10.1371/journal.pone.0141174

**Published:** 2015-10-15

**Authors:** Hamed R.-Tavakoli, Adham Atyabi, Antti Rantanen, Seppo J. Laukka, Samia Nefti-Meziani, Janne Heikkilä

There are errors in the Funding section. The correct funding information is as follows:

This work is supported by Infotech Oulu, Academy of Finland Grant No. 259431, Nokia Scholarships, and The Finnish center of excellence in computational inference research (COIN). The funders had no role in study design, data collection and analysis, decision to publish, or preparation of the manuscript.

Additionally, there is an error in the Competing Interests section. The correct competing interests information is as follows: This work was partially supported by Nokia Scholarships. This does not alter our adherence to all the PLOS ONE policies on sharing data and materials. The publisher apologizes for these errors.
