# Supplementary material for: Predicting the Valence of a Scene from Observers’ Eye Movements
Source: PLoS One. 2015 Sep 25;10(9):e0138198. doi: 10.1371/journal.pone.0138198 (PMC4583411; doi:10.1371/journal.pone.0138198)
Supplement: S1 File — Fig A in S1 File, dimension reduction mechanism. Fig B in S1 File, performance achieved by conventional and evolutionary based decomposition methods. Fig C in S1 File, analysis of individual features using bagging approaches. Fig D in S1 File, analysis of features combinations using bagging approaches. Table A in S1 File, the initial setup of PSO. Table B in S1 File, parameter settings of the variations of PSO approach employed in this study. Table C in S1 File, sample size and feature combination. Table D in S1 File, confusion matrices: mean features and RBF SVM. Table E in S1 File, confusion matrices: mean features and polynomial SVM. Table F in S1 File, confusion matrices: mean features and linear SVM. Table G in S1 File, confusion matrices: individual features and RBF SVM. Table H in S1 File, confusion matrices: individual features and polynomial SVM. Table I in S1 File, confusion matrices: individual features and linear SVM. Table J in S1 File, confusion matrices: feature selection/decomposition and RBF SVM. Table K in S1 File, confusion matrices: feature selection/decomposition and polynomial SVM. Table L in S1 File, confusion matrices: feature selection/decomposition and linear SVM. (PDF) [file pone.0138198.s001.pdf]

Predicting the valence of a scene from observers' eye movements
Supplementary Material

Hamed R.-Tavakoli, Adham Atyabi, Antti Rantanen,
Seppo J. Laukka, Samia Nefti-Meziani, Janne Heikkilä

October, 2014

Dimension Reduction

Dimension Reduction (DR) is a favorable preprocessing stage that is used to address the dimensionality problem of data through feature reduction. It addresses a variety of techniques such as decomposition-based methods, e.g., Common Spatial Pattern (CSP), Principle Component Analysis (PCA), Singular Value Decomposition (SVD).

The general term of dimension reduction can be expanded to feature reduction and feature selection. Feature Reduction (FR), which includes decomposition techniques, is referred to circumstances in which the feature sets are homogeneous and the aim is to identify a subset of this homogeneous data that best represent the underlying pattern. Feature Selection (FS) is referred to circumstances in which the feature set is heterogeneous and contains the features with different natures/types (e.g. frequency, time-frequency, special-temporal, entropy, variance, etc). The aim of feature selection is to identify a sub-combination of feature types that only includes the feature types that have the highest contribution on the detection of underlying patterns.

Sequential feature selection is a conventional approach for identifying a subset of features that best suits the classification performance with two variations of *Sequential Forward Selection (SFS)* and *Sequential Backward Selection (SBS)*. SFS is a bottom-up search approach that gradually adds features down selected by an evaluation function to an empty set S with the condition of minimizing the mean square error (MSE) [1]. The current study utilizes Matlab's 2010b implementation of SFS and SBS (i.e., sequentialfs).

Evolutionary-based dimension reductions such as Genetic Algorithm (GA) and Particle Swarm Optimization (PSO) are alternative approaches used for dimension reduction. While sequential feature selection techniques narrow the number of features by sequential analysis of features using some objective function (e.g. mean square error), evolutionary based techniques such as genetic algorithm and particle swarm optimization (PSO) somehow rely on an evolving population of feature sets. These approaches evolve until converging towards a subset of features that achieve the best classification performance in a mechanism depicted in Figure A.

Genetic Algorithm is an evolutionary approach developed by John Holland in 1975 [2]. GA utilizes operators such as natural selection, crossover, and mutation [3] and been successfully applied to several problems aiming to optimize solution finding process [4]. It takes advantage from Selection, Crossover, and Mutation stages. In Selection stage chromosomes (solution candidates) are ranked according to their fitness with a subset of highly ranked chromosomes being chosen for generating a new generation of chromosomes. In Crossover stage, new generation of chromosomes are evolved from pairs of parenting chromosomes identified in the selection stage. Such a process is facilitated through dividing the parenting chromosomes to two equal half and each parenting chromosome contributing to the generation of the child

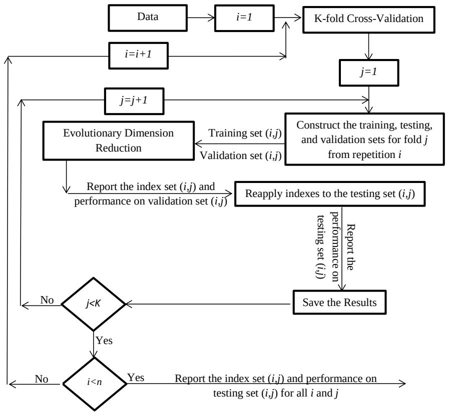

Fig. A. A diagram representing the mechanism proposed for dimension reduction using evolutionary approaches.

via sharing one half of its chromosome. The Mutation stage is the process of introducing randomized changes to parts of a chromosome. This stage is typically utilized in cases where the population is converging towards a local optimum without a chance of generating any better solution due to a lack of genetic diversity. The mutation process can be applied to a single or a sub-set of chromosomes. It is a custom to maintain (repeat) the best performing chromosome, which is known as Elitism. Various implementations of GA have been utilized for dimension reduction, feature selection, and early fusion [5, 6, 7, 8, 9, 10, 11, 12, 13, 14]. A comprehensive review of various evolutionary methods and their comparative performance on a collection of benchmark functions can be found in [15].

Algorithm A represents a pseudocode for the GA-based feature reduction (decomposition). The members of the population (chromosomes) are represented by a binary vector referred to as mask. The mask size is equivalent to the number of feature points in the feature set. The population size and the maximum iteration number are both set to 100. GA-based feature selection uses a procedure akin to algorithm A, except that the binary mask size is to be equal to the number of feature types in the feature set and 0 and 1 values in such mask are to be representative of inclusion and omission of all samples associated to the selected or omitted sample type respectively.

Algorithm A Binary GA pseudo-code for feature reduction

Initialization: Set the population size to 100 and randomly initialize the population by creating binary vectors of the size of the feature set for each of them. Then, evaluate all the members of the population.

repeat

- **Selection:** Sort the population based on performance and eliminate the lesser half.
- **Crossover:** Run a crossover operator (i.e. propose feature combinations) on the remaining population to regenerate a new population.
- **Mutation:** Perform a random mutation (i.e. perturb the feature proposals) over some members which have not improved over time.
- **Validate:** Evaluate all the members of the population (i.e. proposed feature combinations) using train and validation sets.

until (bookmaker > 0.9 or maximum iteration is reached)

Evaluation: Select features suggested by the best member of the population and evaluate the test set.

Particle swarm optimization is an evolutionary approach inspired from animal social behaviors.

Algorithm B PSO pseudecode for feature reduction

Initialization: Randomly initialize the population by creating binary vectors of the size of the feature set for each of them. Then, evaluate all the members of the population using the validation set.
repeat
  Update population: Update the velocity in each particle using (1) and update the binary vectors using (4).
  Evaluation: Evaluate all the members of the population using the validation set.
  Update bests: Update personal-best ($p_{i,j}$) and global-Best (g) of particles using (5) and (6).
until (performance of global-best > 0.9 informedness or the maximum iteration is reached)
Evaluation: Evaluate the test set using the features proposed by the particle which achieved the global-best.

The possible solutions in the search space are modeled using entities called particles. These particles are defined by their velocities $V_{i,j}$ and their positions in the search space $X_{i,j}$ (i represents the particle's index and j represents the dimension in the search space) [16]. Particles in PSO algorithm evolve based on following updating equations:

$$V_{i,j}(t) = w * V_{i,j}(t-1) + C_{i,j} + S_{i,j}, \quad (1)$$

$$C_{i,j} = c_1 r_{1,j} * (p_{i,j}(t-1) - x_{i,j}(t-1)), \quad (2)$$

$$S_{i,j} = c_2 r_{2,j} * (g_{i,j}(t-1) - x_{i,j}(t-1)), \quad (3)$$

$$x_{i,j}(t) = x_{i,j}(t-1) + V_{i,j}(t), \quad (4)$$

where $r_{1,j}$ and $r_{2,j}$ are random values in the range of 0 and 1, c_1 and c_2 are acceleration coefficients employed to control the influences of social and cognitive components. Inertia Weight w is employed to control the influence of previous iteration's velocity (decision) in the current iteration t. Large and small inertia weights causes exploration and exploitation resulting in local-minima avoidance and convergence towards optimum respectively.

In basic PSO, particles memories their best individual and swarm findings. These are referred to as local/personal and global-best solutions. The personal-best solutions, $p_{i,j}$, is the best solution found by each particle and global-best solutions, $g_{i,j}$, is the best performing solution found by the swarm. To update personal and global best solutions, we iteratively apply:

$$p_i(t) = \begin{cases} p_i(t-1) & if \quad f(x_i(t)) >= f(p_i(t-1)) \\ x_i(t) & otherwise. \end{cases} \quad (5)$$

$$g(t) = \arg\min \{f(p_1(t)), f(p_2(t)), ...f(p_s(t))\} . \quad (6)$$

Algorithm B represents a PSO-based feature reduction scheme. Similar to algorithm A members of the population (particles) are defined by a binary vector with the same size as the dimension of feature set. These vectors are referred to as masks. Table A reports the initial parameter adjustment values. Table B presents settings of various PSO based dimension reduction techniques applied in the study. PSO-based feature selection also employs a similar algorithm, where the binary mask size is to be equal to the number of feature types in the feature set and 0 and 1 values in such mask are to be representative of inclusion and omission of all samples associated to the selected or omitted sample type, respectively.

Table A. The initial parameter setup of PSO

Parameters	The Value	The Adjustment Method
c_1	0.5	Fixed Acceleration Coefficient
c_2	0.5	(FAC)
w_1	0.2	Linearly Decreasing Inertia Weight
w_2	1	(LDIW)

Table B. Parameter settings of the variations of PSO approach employed in the study

Approach	Inertia Weight		Acceleration Coefficient		Max iteration	Population size	Details
	W1	W2	C1	C2			
PSO-based decomposition 1	0.2	1	0.5	2.5	100	100	Basic PSO with LDIW and Fix Acceleration Coefficients with 90% decomposition
Mutated PSO-based decomposition 1	0.2	1	0.5	2.5	100	100	Mutation based PSO using LDIW and Fix Acceleration Coefficients with 90% decomposition
Mutated PSO-based decomposition 2	0.2	1	0.5	2.5	100	100	Mutation based PSO using LDIW and Fix Acceleration Coefficients with 50% decomposition
Mutated PSO-based decomposition 3	0.729844	0.729844	0.5	0.5	100	100	Mutation based PSO using Fix inertia weight and Acceleration Coefficients with 90% decomposition

Additional experiments

More details on combination of features

This experiment studies the fusion of features meanwhile employing GA and PSO methods for dimension reduction. First, binary GA and binary PSO are used for sub-selecting the features and training the classifiers. In addition, modified versions of PSO are also considered in order to better assess the contribution of the resulting evolutionary based decomposition methods. These variations address issues such as reduction in the decomposition percentage and mutation of particles that are sub-selecting the features in addition to two different types of parameter settings. The list of the studied approaches are presented in Table B.

Figure B depicts the results of the evolutionary based approaches, SFS, SBS, and the *'Full Set'* as a baseline. The results indicate that the variations of PSO-based decomposition methods show better overall performance in comparison with SFS method while *Mutated PSO-based decomposition 2* method illustrated the best performance among them with only 0.1 bookmaker informedness below the best performing method and less than 0.08 below the full set. The combinations of Polynomial or RBF SVM with either SBS or GA-based decomposition shows no significant difference from the performance achieved by the Full Set. Nonetheless, the overall advantage of the combination of features using SBS and linear SVM is absolutely evident from the results.

The statistical significance analysis of results reveals significant differences ($p = 0.0001 < 0.05$) among dimension reduction approaches applied, i.e., selection, decomposition, none-reduction. However, no significance is observable among classifiers and the interactions of classifiers and dimension reduction approaches ($p = 0.2515 > 0.05$ and $p = 0.7095 > 0.05$ respectively). Further, analysis of significance among the dimension reduction approaches indicated that SBS, GA-based decomposition, and none-reduction (i.e. Full Set) are significantly different from PSO-based decomposition and SFS approach.

Sample size

Despite successful above chance valence recognition, the limited number of training samples could have affected the overall performance of the methods. To address this issue, we utilize ensemble learning approach using bagging with repetition. We generate 100 random manipulations of the training samples

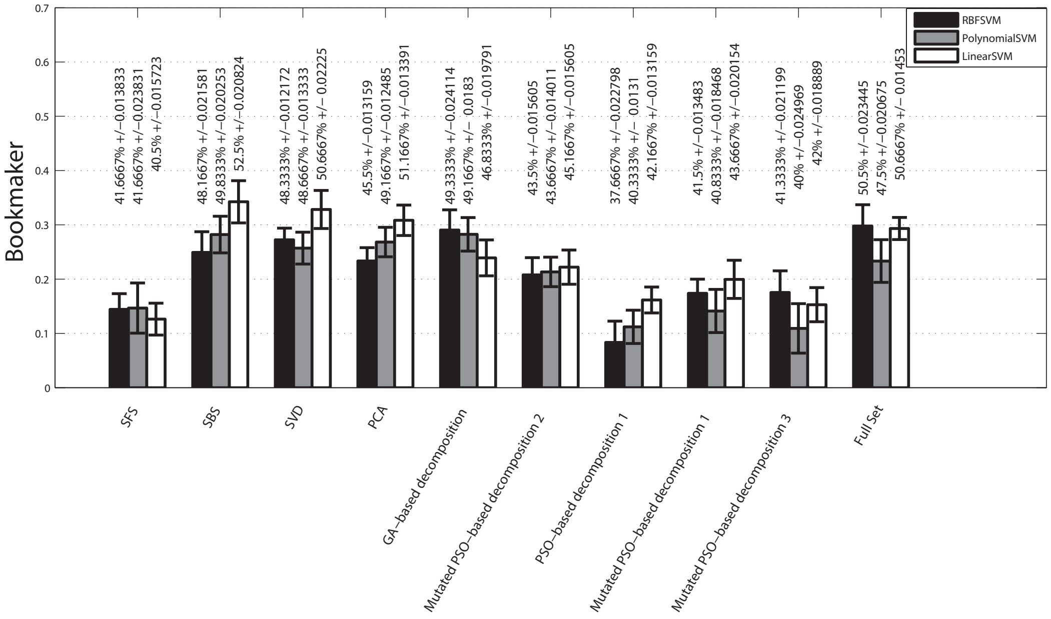

Fig. B. Performance achieved by conventional and evolutionary based decomposition methods.Mean of classification accuracies across folds and repetitions (%) and their associated standard errors are added to each bar as a second measurement unit.

in each fold to generate 100 training sets (so called bags) and use them for training individual SVM classifiers. Afterwards, we assess the test set using each classifier and use the weighted summation operator to derive a decision for each fold. Both baseline and feature combination experiments are studied using this approach. The result of baseline experiment using bagging is summarized in Figure C. It indicates bagging and non-bagging approaches are almost similar. In other words, the classification performance is not necessarily affected by the number of training samples.

The same procedure applied to the combination of features in a mixture of expertise scenario where separate classifiers are trained on one feature type at the time and the overall classification of the test set is computed through summation and weighted summation aggregation. The experiment includes all the previously tested methods as well as a GA based early fusion scheme, which contrarily to the former GA-based decomposition, captures all feature bins of sub-selected features in its reduced feature-set for training the classifiers. Table C summarizes the methods and their abbreviations through the experiments.

The results, as depicted in Figure D, indicate feasibility of GA based early fusion and the combinations of GA based decomposition and bagging technique. The significance analysis of the results indicates a significant difference among dimension reduction approaches ($p = 0 < 0.05$), classifiers ($p = 0.0033 < 0.05$), and the interactions of classifiers and dimension reduction methods ($p = 0.0133 < 0.05$). Further analysis of the results also indicated that GA based feature selection, GA-based decomposition, and mixture of all features are not significantly different from each other while such significance exist between them and variations of bagging and mixture of expertise methods. To summarize, increasing the training samples through either bagging, mixture of expertise, or combination of mixture of expertise and bagging performs similar to the original methods and the classification performance is not necessarily affected by the number of training samples.

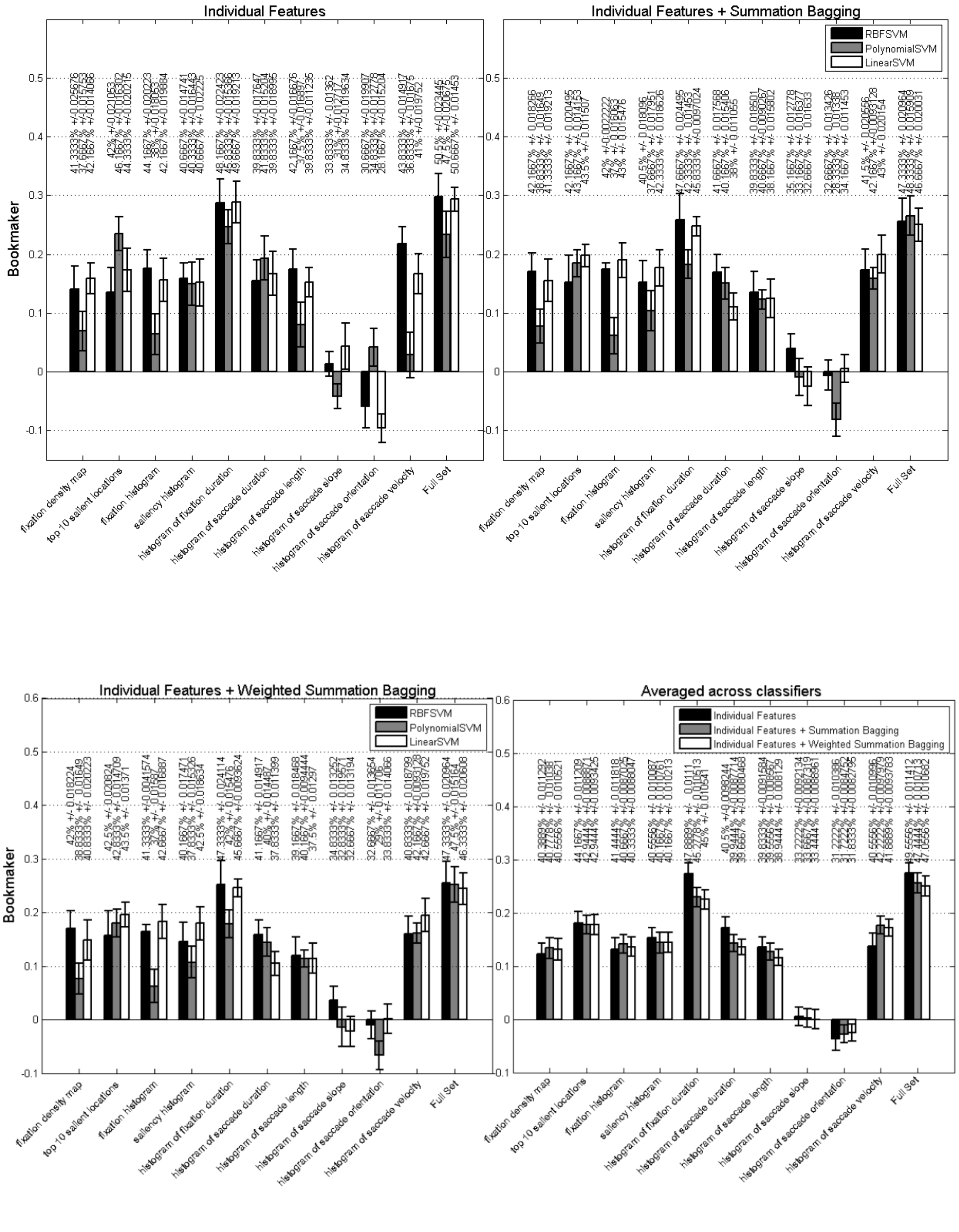

Fig. C. Analysis of individual features using bagging approaches

Table C. Sample size and feature combination: the methods, their abbreviations, and properties.

Approach	Base Approach	Feature Selection	Decomposition	Bagging	Mixture of Expertise	Summation	Weighted Summation
GA-based feature selection	GA	√	x	x	x	x	x
Bagging	Full Set	x	x	√	x	√	x
GA-based decomposition & Bagging	GA	x	√	√	x	x	x
GA-based decomposition & Sum Bagging	GA	x	√	√	x	√	x
GA-based decomposition & Weighted Sum Bagging	GA	x	√	√	x	x	√
Sum Mixture of Expertise	Full Set	x	x	x	√	√	x
Weighted Sum Mixture of Expertise	Full Set	x	x	x	√	x	√
Sum Bagging & Mixture of Expertise	Full Set	x	x	√	√	√	x
Weighted Sum Bagging & Mixture of Expertise	Full Set	x	x	√	√	x	√

Table D. Confusion matrices: mean features and RBF SVM.

(a) mean saccade velocity

Actual \ Prediction	unpleasant	pleasant	neutral
unpl...	11.5	46.0	42.5
pleasant	14.5	47.0	38.5
neutral	16.0	52.0	32.0

(b) mean saccade slope

Actual \ Prediction	unpleasant	pleasant	neutral
unpl...	15.5	39.5	45.0
pleasant	20.5	43.0	36.5
neutral	16.0	30.0	54.0

(c) mean saccade orientation

Actual \ Prediction	unpleasant	pleasant	neutral
unpl...	6.0	51.0	43.0
pleasant	18.0	47.5	36.0
neutral	19.5	49.0	31.5

(d) mean saccade length

Actual \ Prediction	unpleasant	pleasant	neutral
unpl...	22.5	41.0	36.5
pleasant	4.5	40.0	55.5
neutral	3.5	26.0	70.5

(e) mean saccade duration

Actual \ Prediction	unpleasant	pleasant	neutral
unpl...	32.0	49.0	19.0
pleasant	20.5	69.0	10.5
neutral	39.0	46.0	15.0

(f) mean fixation duration

Actual \ Prediction	unpleasant	pleasant	neutral
unpl...	86.0	4.5	9.5
pleasant	68.0	3.0	29.0
neutral	70.0	9.5	20.5

Confusion matrices

Bookmaker Informedness is employed as the main measurement unit for assessing the performance of approaches. Aiming to provide better understanding of the results, the average accuracies and standard errors are also depicted in all of the relevant figures. Although we considered the employed assessment measurement mechanisms and units informative enough, the confusion matrices of the results achieved within each experiment are also provided in this section. The mechanism utilized for reporting the confusion matrices is based on summation of the matrices within folds, repetitions, and if applicable methods and calculating the average accuracy for each component/element of the contingency matrix using following equation:

$$CM_{k,j} = \frac{cm_{k,j}}{\sum_{i=1}^{n} cm_{i,j}} \tag{7}$$

where k and j represent row and column indexes respectively and n is the total number of rows in the contingency matrix (e.g., $n = 3$ in this study).

References

1. Marcano-Cedeno A, Quintanilla-dominguez J, Cortina-Januchs MG, Andina D. Feature selection using Sequential Forward Selection and classification applying Artificial Metaplasticity Neural Network. In: IECON; 2010. p. 2845–2850.

2. Holland H. Adaptation in Natural and Artificial Systems. University of Michigan Press; 1975.

Table E. Confusion matrices: mean features and polynomial SVM.

(a) mean saccade velocity

Actual \ Prediction	unpleasant	pleasant	neutral
unpl...	1.0	76.0	23.0
pleasant	1.5	75.5	23.0
neutral	3.0	75.0	22.0

(b) mean saccade slope

Actual \ Prediction	unpleasant	pleasant	neutral
unpl...	41.0	15.5	43.5
pleasant	50.0	14.0	36.0
neutral	36.5	15.5	48.0

(c) mean saccade orientation

Actual \ Prediction	unpleasant	pleasant	neutral
unpl...	27.0	34.5	38.5
pleasant	39.5	21.0	39.5
neutral	37.0	25.5	37.5

(d) mean saccade length

Actual \ Prediction	unpleasant	pleasant	neutral
unpl...	13.0	30.5	56.5
pleasant	0	24.5	75.5
neutral	0	22.0	78.0

(e) mean saccade duration

Actual \ Prediction	unpleasant	pleasant	neutral
unpl...	9.5	81.5	9.0
pleasant	2.0	93.5	4.5
neutral	9.0	84.5	6.5

(f) mean fixation duration

Actual \ Prediction	unpleasant	pleasant	neutral
unpl...	94.0	5.0	1.0
pleasant	80.5	9.0	10.5
neutral	76.0	13.0	11.0

Table F. Confusion matrices: mean features and linear SVM.

(a) mean saccade velocity

Actual \ Prediction	unpleasant	pleasant	neutral
unpl...	1.0	56.0	34.0
pleasant	11.5	53.5	35.0
neutral	14.0	60.5	25.5

(b) mean saccade slope

Actual \ Prediction	unpleasant	pleasant	neutral
unpl...	3.0	29.5	52.5
pleasant	22.0	29.5	48.5
neutral	17.0	26.0	57.0

(c) mean saccade orientation

Actual \ Prediction	unpleasant	pleasant	neutral
unpl...	17.5	42.0	40.5
pleasant	25.0	42.5	32.5
neutral	25.5	38.5	36.0

(d) mean saccade length

Actual \ Prediction	unpleasant	pleasant	neutral
unpl...	21.5	37.0	41.5
pleasant	0.5	40.0	59.5
neutral	3.5	23.0	73.5

(e) mean saccade duration

Actual \ Prediction	unpleasant	pleasant	neutral
unpl...	31.0	50.0	19.0
pleasant	20.5	69.5	10.0
neutral	38.0	46.0	16.0

(f) mean fixation duration

Actual \ Prediction	unpleasant	pleasant	neutral
unpl...	83.5	5.5	11.0
pleasant	66.5	3.5	30.0
neutral	64.0	12.0	24.0

Table G. Confusion matrices: individual features and RBF SVM.

(a) fixation density map

Actual \ Prediction	unpleasant	pleasant	neutral
unpl...	49.5	31.5	19.0
pleasant	47.5	30.0	22.5
neutral	19.5	36.0	44.5

(b) 10 Salient locations

Actual \ Prediction	unpleasant	pleasant	neutral
unpl...	31.0	9.5	59.5
pleasant	15	38.5	46.5
neutral	25.5	18.0	56.5

(c) fixation histogram

Actual \ Prediction	unpleasant	pleasant	neutral
unpl...	49.5	23.5	27.0
pleasant	38.0	28.5	33.5
neutral	16.0	29.5	54.5

(d) saliency histogram

Actual \ Prediction	unpleasant	pleasant	neutral
unpl...	30.5	29.5	40.0
pleasant	38.0	23.0	39.0
neutral	24.5	7.0	68.5

(e) histogram of fixation duration

Actual \ Prediction	unpleasant	pleasant	neutral
unpl...	25.0	32.0	43.0
pleasant	11.0	39.0	50.0
neutral	5.0	14.5	80.5

(f) histogram of saccade duration

Actual \ Prediction	unpleasant	pleasant	neutral
unpl...	29.0	32.0	39.0
pleasant	34.0	14.0	52.0
neutral	11.0	12.5	76.5

(g) histogram of saccade length

Actual \ Prediction	unpleasant	pleasant	neutral
unpl...	30.0	22.0	48.0
pleasant	26.5	24.5	49.0
neutral	10.5	17.5	72.0

(h) histogram of saccade slope

Actual \ Prediction	unpleasant	pleasant	neutral
unpl...	13.0	52.0	35.0
pleasant	14.5	53.0	32.5
neutral	8.5	56.0	35.5

(i) histogram of saccade orientation

Actual \ Prediction	unpleasant	pleasant	neutral
unpl...	22.0	40.5	37.5
pleasant	31.5	36.5	32.0
neutral	37.5	29.0	33.5

(j) histogram of saccade velocity

Actual \ Prediction	unpleasant	pleasant	neutral
unpl...	33.0	29.5	37.5
pleasant	35.0	20.5	44.5
neutral	13.0	9.0	78.0

(k) full set

Actual \ Prediction	unpleasant	pleasant	neutral
unpl...	23.5	8.5	68.0
pleasant	10.0	40.0	50.0
neutral	3.0	9.0	88.5

Table H. Confusion matrices: individual features and polynomial SVM.

(a) fixation density map

Actual	Prediction		
	unpleasant	pleasant	neutral
unpl...	23.0	77.0	00.0
pleasant	25.5	73.5	1.0
neutral	20.0	63.5	16.5

(b) 10 Salient locations

Actual	Prediction		
	unpleasant	pleasant	neutral
unpl...	31.0	21.5	47.5
pleasant	10.0	60.0	30.0
neutral	17.0	35.5	47.5

(c) fixation histogram

Actual	Prediction		
	unpleasant	pleasant	neutral
unpl...	12.5	84.5	3.0
pleasant	11.0	78.5	10.5
neutral	5.0	72.0	23.0

(d) saliency histogram

Actual	Prediction		
	unpleasant	pleasant	neutral
unpl...	54.0	13.0	33.0
pleasant	56.5	11.5	32.0
neutral	37.5	7.0	55.5

(e) histogram of fixation duration

Actual	Prediction		
	unpleasant	pleasant	neutral
unpl...	17.0	17.5	65.5
pleasant	7.5	29.0	63.5
neutral	1.5	7.0	91.5

(f) histogram of saccade duration

Actual	Prediction		
	unpleasant	pleasant	neutral
unpl...	36.5	31.5	32.0
pleasant	38.0	16.0	46.0
neutral	15.0	12.0	73.0

(g) histogram of saccade length

Actual	Prediction		
	unpleasant	pleasant	neutral
unpl...	43.0	36.0	21.0
pleasant	44.5	28.5	27.0
neutral	27.0	32.0	41.0

(h) histogram of saccade slope

Actual	Prediction		
	unpleasant	pleasant	neutral
unpl...	14.5	53.5	32.0
pleasant	14.5	53.0	32.5
neutral	16.0	58.5	25.5

(i) histogram of saccade orientation

Actual	Prediction		
	unpleasant	pleasant	neutral
unpl...	19.0	49.5	31.5
pleasant	20.5	62.0	17.5
neutral	31.0	45.5	23.5

(j) histogram of saccade velocity

Actual	Prediction		
	unpleasant	pleasant	neutral
unpl...	35.5	54.5	10.0
pleasant	53.0	39.5	8.0
neutral	24.5	39.5	36.0

(k) full set

Actual	Prediction		
	unpleasant	pleasant	neutral
unpl...	20.0	23.0	57.0
pleasant	6.0	53.0	41.0
neutral	6.5	24.0	69.5

Table I. Confusion matrices: individual features and linear SVM.

(a) fixation density map

Actual	Prediction		
	unpleasant	pleasant	neutral
unpl...	45.5	34.0	20.5
pleasant	41.0	35.5	23.5
neutral	16.0	38.5	45.5

(b) 10 Salient locations

Actual	Prediction		
	unpleasant	pleasant	neutral
unpl...	33.0	14.5	53.5
pleasant	20.5	43.5	36.0
neutral	28.0	15.5	56.5

(c) fixation histogram

Actual	Prediction		
	unpleasant	pleasant	neutral
unpl...	51.0	23.0	26.0
pleasant	38.5	26.0	35.5
neutral	17.0	33.5	49.5

(d) saliency histogram

Actual	Prediction		
	unpleasant	pleasant	neutral
unpl...	35.0	29.0	36.0
pleasant	42.0	24.0	34.0
neutral	31.0	6.0	63.0

(e) histogram of fixation duration

Actual	Prediction		
	unpleasant	pleasant	neutral
unpl...	29.5	29.0	41.5
pleasant	8.5	42.0	49.5
neutral	8.5	14.0	77.5

(f) histogram of saccade duration

Actual	Prediction		
	unpleasant	pleasant	neutral
unpl...	23.0	35.5	41.5
pleasant	31.5	19.0	49.5
neutral	7.0	15.5	77.5

(g) histogram of saccade length

Actual	Prediction		
	unpleasant	pleasant	neutral
unpl...	25.0	25.0	50.0
pleasant	30.0	21.5	48.5
neutral	9.5	17.5	73.0

(h) histogram of saccade slope

Actual	Prediction		
	unpleasant	pleasant	neutral
unpl...	8.5	59.0	32.5
pleasant	7.0	70.5	22.5
neutral	6.0	68.5	25.5

(i) histogram of saccade orientation

Actual	Prediction		
	unpleasant	pleasant	neutral
unpl...	22.5	35.0	42.5
pleasant	35.5	34.5	30.0
neutral	47.0	25.5	27.5

(j) histogram of saccade velocity

Actual	Prediction		
	unpleasant	pleasant	neutral
unpl...	33.0	31.5	35.5
pleasant	38.0	19.0	43.0
neutral	16.5	12.5	71.0

(k) full set

Actual	Prediction		
	unpleasant	pleasant	neutral
unpl...	37.5	15.0	47.5
pleasant	22.5	40.5	37.0
neutral	13.0	13.0	74.0

Table J. Confusion matrices: feature selection/decomposition and RBF SVM.

(a) SFS

	Prediction		
Actual	unpleasant	pleasant	neutral
unpl...	33.5	32.5	34.0
pleasant	32.0	40.0	28.0
neutral	19.5	29.0	51.5

(b) SBS

	Prediction		
Actual	unpleasant	pleasant	neutral
unpl...	38.0	12.5	49.5
pleasant	20.5	39.5	40.0
neutral	16.5	16.5	67.0

(c) SVD

	Prediction		
Actual	unpleasant	pleasant	neutral
unpl...	24.0	12.5	63.5
pleasant	16.0	36.5	47.5
neutral	3.5	12.0	84.5

(d) PCA

	Prediction		
Actual	unpleasant	pleasant	neutral
unpl...	21.0	16.0	63.0
pleasant	18.5	35.5	46.0
neutral	6.0	14.0	80.0

(e) GA-based decomposition

	Prediction		
Actual	unpleasant	pleasant	neutral
unpl...	28.0	20.0	52.0
pleasant	16.0	45.5	38.5
neutral	7.5	18.0	74.5

(f) mutated PSO-based decomposition 2

	Prediction		
Actual	unpleasant	pleasant	neutral
unpl...	26.0	30.0	44.0
pleasant	21.5	38.0	40.5
neutral	12.0	21.5	66.5

(g) GA-based feature selection

	Prediction		
Actual	unpleasant	pleasant	neutral
unpl...	32.0	28.0	40.0
pleasant	26.0	37.0	37.0
neutral	12.0	24.0	64.0

(h) full set

	Prediction		
Actual	unpleasant	pleasant	neutral
unpl...	23.5	8.5	68.0
pleasant	10.0	40.0	50.0
neutral	3.0	9.0	88.0

Table K. Confusion matrices: feature selection/decomposition and polynomial SVM.

(a) SFS

	Prediction		
Actual	unpleasant	pleasant	neutral
unpl...	35.5	27.0	37.5
pleasant	31.0	36.5	32.5
neutral	23.0	24.0	53.0

(b) SBS

	Prediction		
Actual	unpleasant	pleasant	neutral
unpl...	31.5	25.0	43.5
pleasant	11.5	55.0	33.5
neutral	12.5	24.5	63.0

(c) SVD

	Prediction		
Actual	unpleasant	pleasant	neutral
unpl...	31.5	21.5	47.0
pleasant	7.0	50.0	43.0
neutral	8.5	27.0	64.5

(d) PCA

	Prediction		
Actual	unpleasant	pleasant	neutral
unpl...	21.0	31.0	48.0
pleasant	2.5	61.0	36.5
neutral	5.5	29.0	65.5

(e) GA-based decomposition

	Prediction		
Actual	unpleasant	pleasant	neutral
unpl...	26.5	28.5	45.0
pleasant	8.0	53.0	39.0
neutral	8.5	23.5	68.0

(f) mutated PSO-based decomposition 2

	Prediction		
Actual	unpleasant	pleasant	neutral
unpl...	29.0	35.5	35.5
pleasant	27.5	43.5	29.0
neutral	15.0	26.5	58.5

(g) GA-based feature selection

	Prediction		
Actual	unpleasant	pleasant	neutral
unpl...	36.0	32.0	32.0
pleasant	24.5	42.0	33.5
neutral	15.0	20.0	65.0

(h) full set

	Prediction		
Actual	unpleasant	pleasant	neutral
unpl...	20.0	23.0	57.0
pleasant	6.0	53.0	41.0
neutral	6.5	24.0	69.5

Table L. Confusion matrices: feature selection/decomposition and linear SVM.

(a) SFS

	Prediction		
Actual	unpleasant	pleasant	neutral
unpl...	33.5	33.0	33.5
pleasant	30.5	41.0	28.5
neutral	23.0	30.0	47.0

(b) SBS

	Prediction		
Actual	unpleasant	pleasant	neutral
unpl...	40.5	20.0	39.5
pleasant	21.0	44.0	35.0
neutral	12.0	15.0	73.0

(c) SVD

	Prediction		
Actual	unpleasant	pleasant	neutral
unpl...	33.0	17.5	49.5
pleasant	20.0	39.0	41.0
neutral	9.0	11.0	80.0

(d) PCA

	Prediction		
Actual	unpleasant	pleasant	neutral
unpl...	37.0	16.5	46.5
pleasant	19.5	43.5	37.0
neutral	10.5	16.5	73.0

(e) GA-based decomposition

	Prediction		
Actual	unpleasant	pleasant	neutral
unpl...	38.0	16.5	45.5
pleasant	25.0	35.5	39.5
neutral	17.0	16.0	67.0

(f) mutated PSO-based decomposition 2

	Prediction		
Actual	unpleasant	pleasant	neutral
unpl...	27.5	31.0	41.5
pleasant	26.5	31.5	42.0
neutral	10.5	13.0	76.5

(g) GA-based feature selection

	Prediction		
Actual	unpleasant	pleasant	neutral
unpl...	34.0	25.0	41.0
pleasant	29.5	40.0	30.5
neutral	16.0	22.0	62.0

(h) full set

	Prediction		
Actual	unpleasant	pleasant	neutral
unpl...	37.5	15.0	47.5
pleasant	22.5	40.5	37.0
neutral	13.0	13.0	74.0

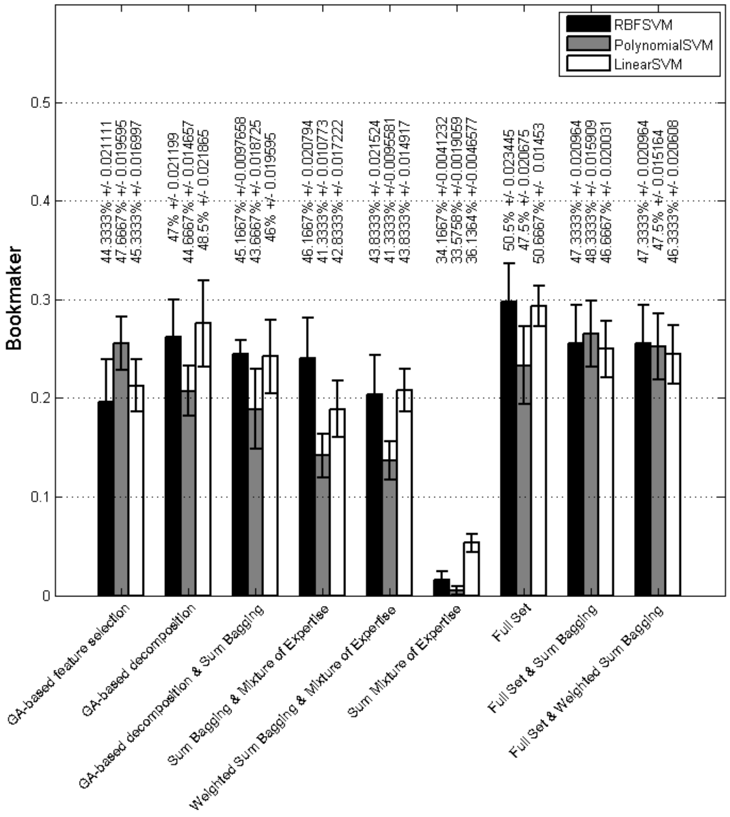

Fig. D. Analysis of features combinations using bagging approaches.

3. Han KM. Collision Free Path Planning Algorithms for Robot Navigation Problem. Department of Electrical and Computer Engineering; 2007.

4. Konar A. Computational Intelligence principles, techniques and applications. Springer Verlag; 2005.

5. Yom-Tov E, Inbar GF. Feature selection for the classification of movements from single movement-related potentials. Neural Systems and Rehabilitation Engineering, IEEE Transactions on. 2002;10(3).

6. Sabeti M, Boostani R, Katebi SD. A New Approach to Classify the Schizophrenic and Normal Subjects by Finding the Best Channels and Frequency Bands. In: Digital Signal Processing; 2007. p. 123–126.

7. Ito Si, Mitsukura Y, Fukumi M, Cao J. Method for detecting music to match the user's mood in prefrontal cortex electroencephalogram activity based on individual characteristics. In: IEEE International Conference on Systems, Man and Cybernetics; 2007. p. 2640–2644.

8. Al-Ani A. A Dependency-based Search Strategy for Feature Selection. Expert Systems with Applications. 2009;36(10).

9. Sanei S, Chambers JA. EEG Signal Processing. John Wiley & Sons, Ltd.; 2007.

10. Dias NS, Jacinto LR, Mendes PM, Correia JH. Feature down-selection in Brain-Computer Interfaces. In: Neural Engineering; 2009. p. 323–326.

11. Zhiping H, Guangming C, Cheng C, He X, Jiacai Z. A new EEG feature selection method for self-paced brain-computer interface. In: Intelligent Systems Design and Applications; 2010. p. 845–849.

12. Dobrea DM, Dobrea MC, Sirbu A. GA methods for selecting the proper EEG individual spectral bands limits. In: Applied Sciences in Biomedical and Communication Technologies (ISABEL), 2010 3rd International Symposium on; 2010. p. 1–6.

13. Atyabi A, Luerssen M, Fitzgibbon S, Powers DMW. Evolutionary feature selection and electrode reduction for EEG classification. In: Evolutionary Computation; 2012. p. 1–8.

14. Li X, Wang Y, Song J, Shan JA. Research on Classification Method of Combining Support Vector Machine and Genetic Algorithm for Motor Imager EEG. Journal of Computational Information Systems. 2012;7(11).

15. Wahab MNA, Nefti-Meziani S, Atyabi A. A Comprehensive Review of Swarm Optimization Algorithms. PLoS ONE. 2015;10(5).

16. Atyabi A, Luerssen MH, Powers DMW. PSO-based dimension reduction of EEG recordings: Implications for subject transfer in BCI. Neurocomputing. 2013;119(0).
